# Computational study on a puzzle in the biosynthetic pathway of anthocyanin: Why is an enzymatic oxidation/ reduction process required for a simple tautomerization?

**DOI:** 10.1371/journal.pone.0198944

**Published:** 2018-06-13

**Authors:** Hajime Sato, Chao Wang, Mami Yamazaki, Kazuki Saito, Masanobu Uchiyama

**Affiliations:** 1 Graduate School of Pharmaceutical Sciences, Chiba University, 1-8-1 Inohana, Chuo-ku, Chiba 260-8675, Japan; 2 Elements Chemistry Laboratory, RIKEN, and RIKEN Center for Sustainable Resource Science (Wako campus) 2-1 Hirosawa, Wako-shi, Saitama 351-0198, Japan; 3 Graduate School of Pharmaceutical Sciences, The University of Tokyo, 7-3-1 Hongo, Bunkyo-ku, Tokyo 113-0033, Japan; 4 RIKEN Center for Sustainable Resource Science (Yokohama campus) 1-7-22 Suehiro-cho, Tsurumi-ku, Yokohama 230-0045, Japan; Universidade de Lisboa Instituto Superior de Agronomia, PORTUGAL

## Abstract

In the late stage of anthocyanin biosynthesis, dihydroflavonol reductase (DFR) and anthocyanidin synthase (ANS) mediate a formal tautomerization. However, such oxidation/reduction process requires high energy and appears to be unnecessary, as the oxidation state does not change during the transformation. Thus, a non-enzymatic pathway of tautomerization has also been proposed. To resolve the long-standing issue of whether this non-enzymatic pathway is the main contributor for the biosynthesis, we carried out density functional theory (DFT) calculations to examine this non-enzymatic pathway from dihydroflavonol to anthocyanidin. We show here that the activation barriers for the proposed non-enzymatic tautomerization are too high to enable the reaction to proceed under normal aqueous conditions in plants. The calculations also explain the experimentally observed requirement for acidic conditions during the final step of conversion of 2-flaven-3,4-diol to anthocyanidin; a thermodynamically and kinetically favorable concerted pathway can operate under these conditions.

## Introduction

**Fig 1:**
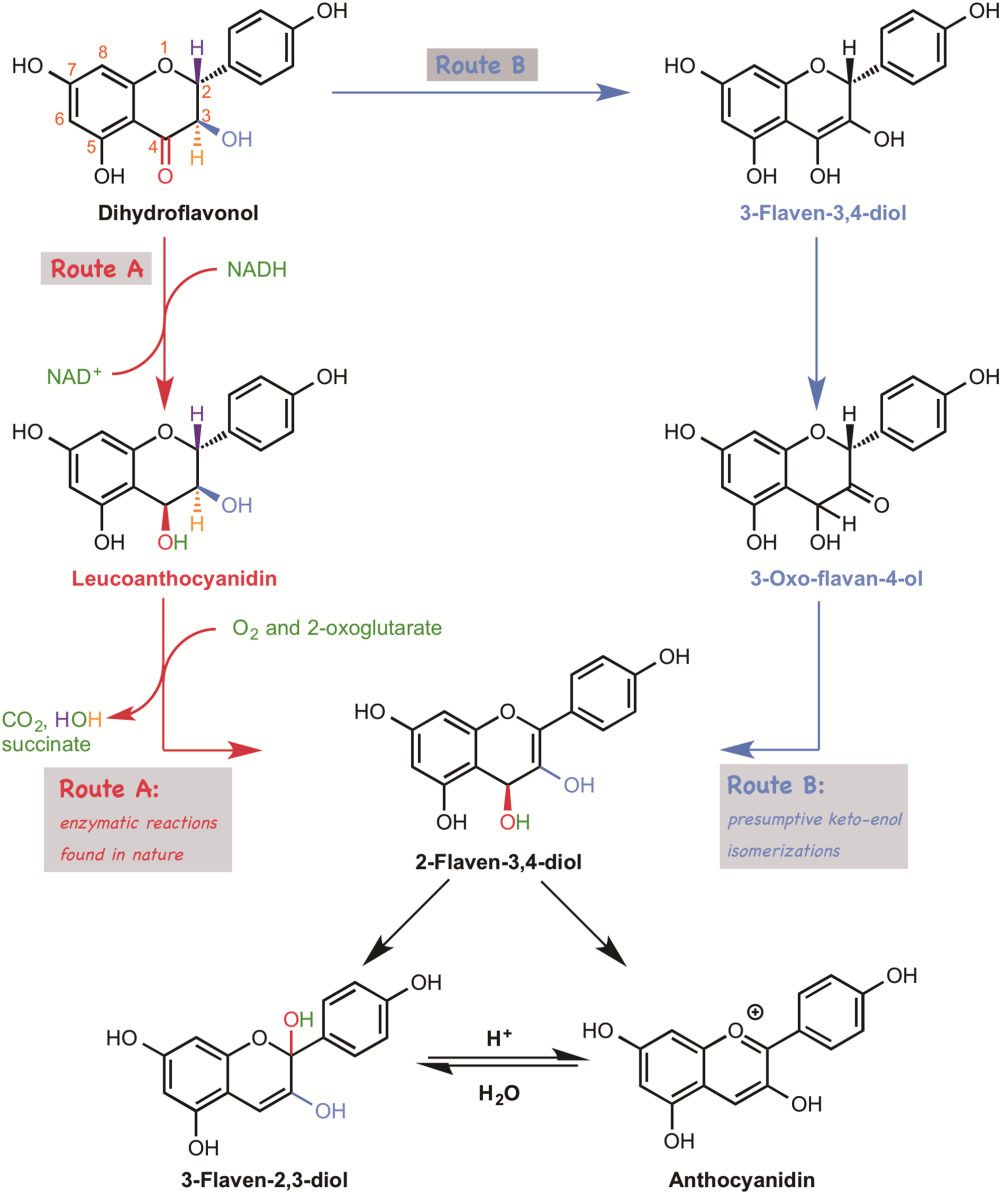
Two possible routes from dihydroflavonol to anthocyanidin. 2-Flaven-3,4-diol has not been isolated because of its instability under acidic conditions. (2R, 3S, 4S)-cis-Leucoanthocyanidine was confirmed to be the enzymatic product of DFR and to act as a substrate of ANS.

Anthocyanins, an important plant secondary metabolites, contribute to the diversity of colored pigments in plants, especially in flowers and fruits, and also act as photo-protectants, [1] visual signals [2] for insects to promote pollination, and antioxidants. [3–6] Although anthocyanin biosynthesis has been extensively studied, [7, 8] some details still remain unclear. In the late stage of anthocyanin biosynthesis, oxidizing and reducing enzymes appear to be necessary for the conversion of dihydroflavonol to anthocyanidin [9, 10] (Fig 1 Route A).

As this conversion is formally a tautomerization, and does not involve a change of oxidation state, a high energy-consumptive oxidation/reduction pathway seems unnecessary. Consequently, a non-enzymatic simple tautomerization pathway has also been proposed (Fig 1 Route B). This is a long-standing issue. The enzymes involved, dihydroflavonol reductase (DFR) [11] and anthocyanidin synthase (ANS), [12–14] were isolated and characterized about a quarter of a century ago. On the other hand, there is no evidence that would rule out a non-enzymatic pathway. ANS is an oxidizing enzyme that catalyzes dehydrogenation at the C-2 position of leucoanthocyanidin, followed by dehydration to afford anthocyanidin. This activity has been confirmed in vitro by using recombinant ANS. [12–14] ANS requires Fe^2+^, 2-oxoglutarate, molecular oxygen, and ascorbate as cofactors, and also needs acidic conditions (pH 1-5) after the enzymatic reaction to generate anthocyanidin. [13, 14] Targeted gene-deletion experiments [5] have confirmed that loss of ANS or DFR essentially abrogates anthocyanidin synthesis, supporting the idea that both DFR and ANS are essential for anthocyanin biosynthesis. However, it is a mystery why the simple non-enzymatic pathway does not operate in anthocyanin biosynthesis. This is an important issue, because a deep mechanistic understanding is a prerequisite for rational design to fulfil modern requirements for the efficient preparation of highly purified anthocyanin or modified anthocyanins. In biochemical reactions, it sometimes seems intuitively clear that an alternative reaction route is less favorable. However, such predictions are just qualitative, and cannot rule out the possibility that the alternative route can proceed spontaneously. Recently, computational chemistry has been extensively used for the mechanistic investigation of natural products biosynthesis, such as terpene cyclization reaction of cycloocatin, [15, 16] and Diels-Alder reaction of heronamide A. [17] This theoretical approach to the study of biosynthesis provides detailed and quantitative information about reaction mechanisms and intermediates, which is helpful for design of further experiments or for engineering the enzymes involved. Therefore, in this work, we conducted a comprehensive computational/theoretical study on the non-enzymatic tautomerization process. Our results indicate that the simple non-enzymatic pathway is energetically much less favorable.

## Methods

All calculations were performed with Gaussian 09 [18] and GRRM11 [19–23] programs. Structure optimization and frequency calculation were done with the M06-2X/6-31G(d,p) method. [24, 25] Solvation was evaluated by the self-consistent reaction field (SCRF) method using the polarizable continuum model (PCM). Single point energy was calculated at the MP2/6-311++G(d,p) level based on the M06-2X-optimized structure, since M06-2X is known to be inappropriate for describing the relative energies of proton transfer reactions. [26] Relative Gibbs free energy energies (ΔG*rel*) based on single point energy at the MP2 level and frequency calculation at the M06-2X level are given for all discussions.

## Results and discussion

**Fig 2:**
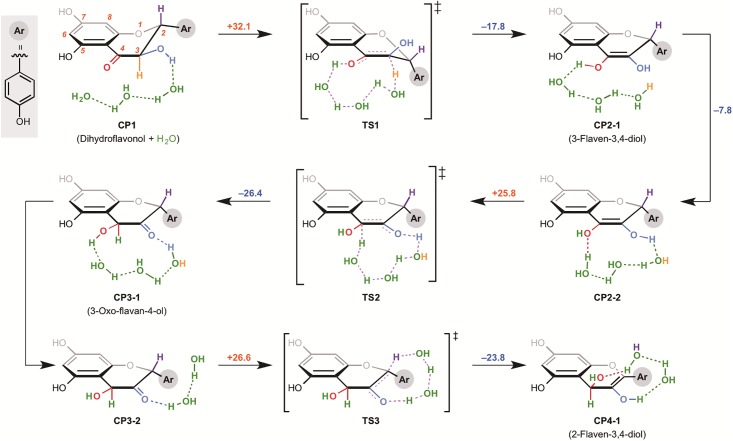
Two possible routes from dihydroflavonol to anthocyanidin.

The optimized structures (without any symmetry assumptions) and energies of all CPs (complexes), TSs (Transition States), and the product in the conversion of dihydroflavonol to 2-flaven-3,4-diol are shown in Fig 2.

The results of several examinations indicated that the conversion of dihydroflavonol to 3-flaven-3,4-diol requires very high activation energy (31.8 kcal/mol). It is well known that reaction at room temperature can take place only when the activation energy is lower than 20-25 kcal/mol, and therefore, this step cannot take place in plants. The enediol structure of 3-flaven-3,4-diol is quite unstable, because this enediol is adjacent to the electron-rich aromatic A ring. Therefore, this step is endothermic. After the interconversion from CP2-1 to CP2-2 with 8.6 kcal/mol exothermicity, another keto-enol isomerization takes place mediated by H_2_O molecules. Thus, we could successfully locate the non-enzymatic tautomerization process, which involves two sequential keto-enol isomerizations, both mediated by H_2_O molecules. However, the computational results indicated that the non-enzymatic pathway is energetically very unfavorable in plants, since the transition states TS1 and TS2 are very unstable and the activation energies are insurmountably high (31.8 and 27.3 kcal/mol, respectively) under the conditions present in plants; in addition, both steps are endothermic. However, the question remains as to why plants would have evolved enzymes catalyzing these reduction-oxidation reactions instead of enzymes catalyzing the simple tautomerization. To address this intriguing question, we investigated whether there are redox-neutral enzymes which can catalyze this type of tautomerization by searching all enzymatic reactions registered in the KEGG database, [27, 28] using the program ‘E-zyme2’ [29] However, only redox enzymes were found, i.e. K13082; DFR, K05277: leucoanthocyanidin dioxygenase, K13261: CYP93A1, K13265: vestitone reductase, K07409: CYP1A2. Thus, no redox-neutral enzymes that can catalyze this type of tautomerization reaction are currently known. Nevertheless, this does not rule out the possibility that biology took the option of simple tautomerization during evolution. Therefore, as a different approach, we also computed a general acid- or base-catalyzed enzymatic pathway (see supporting information). The calculations suggest that such tautomerization by acid or base (including protein) is unfavorable due to the low reactivity of dihydroflavonol. From the viewpoint of plant physiology, plant cells need an efficient mechanism for redox recycling of NAD(P)H, because of their highly active metabolism, including photosynthesis and energy metabolism [30] This requirement for rapid recycling of NAD(P)H might have favored evolution of an anthocyanin biosynthetic pathway involving oxidation of NAD(P)H. In other words, the combined fundamental chemistry and plant biology requirements could be the reason why the apparently wasteful reduction-oxidation route evolved for the synthesis of anthocyanins.

**Fig 3:**
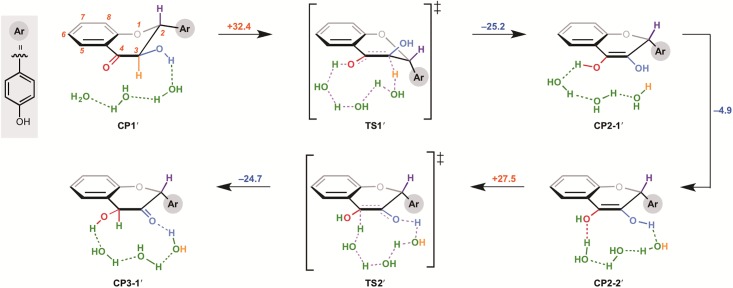
Calculation results for conversion of dihydroflavonol (CP1) to 2-flaven-3,4-diol (CP4-1) via route B in a neutral aqueous environment.

**Fig 4:**
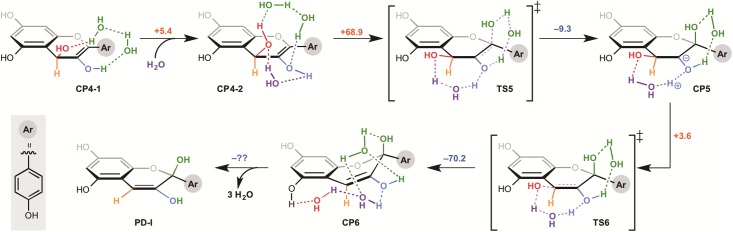
The effect of 2-hydroxyl group on the A ring: conversion of 5,7-dehydroxydihydroflavonol (CP1’) to 3-oxo-flavan-4-ol (CP3-1’).

We then investigated the influence of the two hydroxyl groups of the A ring on the activation energies of the non-enzymatic tautomerization. Firstly, we calculated a presumptive pathway, shown in Fig 3, for an artificial substrate without the 5,7-hydroxyl groups of dihydroflavonol. Surprisingly the activation energy of the formation of corresponding enediol compound was not changed dramatically in comparison to that for dihydroflavonol. The stabilization energy was increased by 4.3 kcal/mol, supporting our hypothesis that the electron-rich A ring is responsible for the instability of 3-flaven-3,4-diol. However, this pathway still has a high activation barrier, suggesting that the reaction is unlikely to occur at ambient temperature. On the other hand, the 5,7-hydroxyl groups do not influence the second enolization step. We also investigated various other possible reaction pathways for the conversion of 2-flaven-3,4-diol to anthocyanidin. The results indicated that the reaction pathway of 1,3-transposition of the hydroxyl group in aqueous media under neutral conditions (Fig 4) is the most probable. In this reaction, 1,3-transposition of the hydroxyl group from C-4 to C-2 proceeds in two steps through TS5 (addition of one molecule of water at the C-2 position) and TS6 (elimination of the OH group at C-4 to form a double bond). However, the activation energy of the first step is 74.9 kcal/mol, which is far too high for the reaction to occur under these conditions. In addition, CP5 is very unstable and hence the reverse reaction should proceed preferentially. Thus, we concluded that formal 1,3-transposition of the hydroxyl group of 2-flaven-3,4-diol does not occur under neutral conditions.This is consistent with the experimental result of ANS assay in vitro, suggesting that the formation of anthocyanidin requires acidification (pH 1-5) after the enzyme reaction.

**Fig 5:**
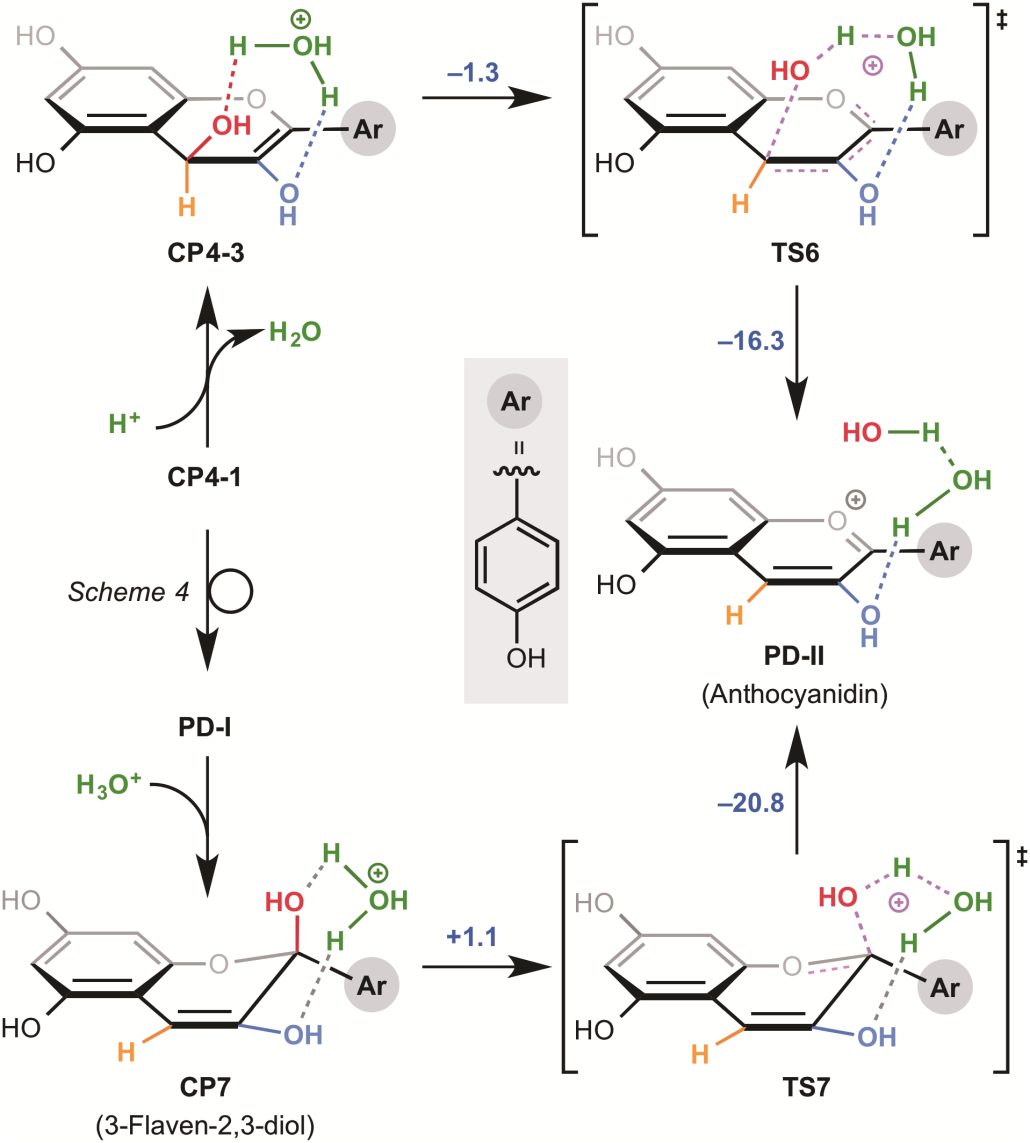
Calculation results for the late stage of the biosynthesis of anthocyanidin in a neutral aqueous environment.

Finally, we re-investigated this step under acidic conditions for the formation of anthocyanidin. The energy profiles at the same level of theory are shown in Fig 5, in which, 2-flaven-3,4-diol is directly converted to anthocyanidin. This transformation takes place as a single event under the acidic conditions. The activated 4-OH of CP4-3 and 2-OH of CP7 are immediately protonated and readily eliminated, respectively, to form anthocyanidin. The activation free energy from CP7 is quite low, and the process from CP4-3 is a barrier-less transformation, suggesting that spontaneous reaction would be expected both thermodynamically and kinetically. Thus, both our calculations and reported experimental results support the idea that acidic conditions or activation of 4-OH of 2-flaven-3,4-diol are necessary to obtain anthocyanidin as a product in the biosynthetic pathway; the direct transformation pathway of 2-flaven-3,4-diol into 3-flaven-2,3-diol at cytosolic pH is ruled out, and 3-flaven-2,3-diol is formed after the formation of anthocyanidin. This may be due to the low leaving ability of 4-OH of CP4-3 and 2-OH of CP7. Instead, we suggest an unprecedented pathway in which 2-flaven-3,4-diol is directly converted to anthocyanidin under acidic conditions. This is also consistent with the fact that in vitro assay of ANS requires acidic conditions for anthocyanidin formation. Thus, the reaction may take place in acidic environments such as plant vacuoles, or alternatively there may be an enzyme that can catalyze the conversion of 3-flaven-2,3-diol to anthocyanidin.

In summary, the results of our computational study provided new insights of anthocyanin biosynthesis, indicating that the activation barriers in non-enzymatic pathways are too high to overcome in the plant cellular environment. We also simulated a hypothetical enzymatic tautomerization pathway, but found that this also requires very high activation energy, and would be unfavorable due to the low reactivity of the substrate, dihydroflavonol, and the low acidity of 2-H of dihydroflavonol. This may be the reason why plants evolved an enzymatic pathway involving reduction-oxidation to achieve anthocyanin production, even though it is highly energy-consumptive. We also investigated the reason why ANS requires acidic conditions for the generation of anthocyanidin. Our calculations indicate that the widely accepted stepwise pathway is actually unfavorable under neutral conditions, and a concerted pathway in which 2-flaven-3,4-diol is directly converted to anthocyanidin is preferred both thermodynamically and kinetically under acidic conditions. This is consistent with the previously reported experimental finding that formation of 3-flaven-2,3-diol was not observed. Our present work shows that computational studies can throw new light on not only terpene cyclization, but also flavonoid biosynthetic mechanisms, providing new ideas for experimental study.

## Supporting information

S1 File Supporting data. General Methods, Energy Profiles, Cartesian Coordinates, Hypothetical Acid-Catalyzed Enzymatic Reaction (S1 Fig), Hypothetical Base-Catalyzed Enzymatic Reaction (S2 Fig), References.
(DOCX)

## References

[1] Feild TS, Lee DW, Holbrook NM. Why Leaves Turn Red in Autumn. The Role of Anthocyanins in Senescing Leaves of Red-Osier Dogwood. Plant Physiol. 2001;127: 566–574. doi: 10.1104/pp.010063 11598230 PMC125091

[2] Wang H, Conchou L, Bessière JM, Cazals G, Schatz B, Imbert E. Flower color polymorphism in Iris lutescens (Iridaceae): Biochemical analyses in light of plant-insect interactions. Phytochemistry. Elsevier Ltd; 2013;94: 123–134. doi: 10.1016/j.phytochem.2013.05.007 10.1016/j.phytochem.2013.05.007 23790644

[3] Nijveldt R. J., van Nood E., van Hoorn D. E. C., Boelens P. G., van Norren K., van Leeuwen P. A. M. Flavonoids a review of probable mechanisms of action. Am. J. Clin. Nutr., 74, 418–425 (2001). doi: 10.1093/ajcn/74.4.418 11566638 10.1093/ajcn/74.4.418

[4] Fauconneau B, Waffo-Teguo P, Huguet F, Barrier L, Decendit A, Merillon JM. Comparative study of radical scavenger and antioxidant properties of phenolic compounds from Vitis vinifera cell cultures using in vitro tests. Life Sci. 1997;61: 2103–2110. doi: 10.1016/S0024-3205(97)00883-7 9395251 10.1016/s0024-3205(97)00883-7

[5] Tohge T, Matsui K, Ohme-Takagi M, Yamazaki M, Saito K. Enhanced radical scavenging activity of genetically modified Arabidopsis seeds. Biotechnol Lett. 2005;27: 297–303. doi: 10.1007/s10529-005-0683-7 15834789 10.1007/s10529-005-0683-7

[6] Butelli E, Titta L, Giorgio M, Mock HP, Matros A, Peterek S, et al Enrichment of tomato fruit with health-promoting anthocyanins by expression of select transcription factors. Nat Biotechnol. 2008;26: 1301–1308. doi: 10.1038/nbt.1506 18953354 10.1038/nbt.1506

[7] Springob K, Nakajima J, Yamazaki M, Saito K. Recent advances in the biosynthesis and accumulation of anthocyanins. Nat Prod Rep. 2003;20: 288 doi: 10.1039/b109542k 12828368 10.1039/b109542k

[8] Kurepa J, Nakabayashi R, Paunesku T, Suzuki M, Saito K, Woloschak GE, et al Direct isolation of flavonoids from plants using ultra-small anatase TiO2 nanoparticles. Plant J. 2014;77: 443–453. doi: 10.1111/tpj.12361 24147867 10.1111/tpj.12361 PMC3935720

[9] Saito K, Yamazaki M. Biochemistry and molecular biology of the late-stage of biosynthesis of anthocyanin: Lessons from Perilla frutescens as a model plant. New Phytol. 2002;155: 9–23. doi: 10.1046/j.1469-8137.2002.00440.x 10.1046/j.1469-8137.2002.00440.x 33873294

[10] Saito K, Yonekura-sakakibara K, Nakabayashi R, Higashi Y. Plant Physiology and Biochemistry The fl avonoid biosynthetic pathway in Arabidopsis?: Structural and genetic diversity. Plant Physiol Biochem. 2013;72: 21–34. 23473981 10.1016/j.plaphy.2013.02.001

[11] Gong ZZ, Yamazaki M, Sugiyama M, Tanaka Y, Saito K. Cloning and molecular analysis of structural genes involved in anthocyanin biosynthesis and expressed in a forma-specific manner in Perilla frutescens. Plant Mol Biol. 1997;35: 915–927. doi: 10.1023/A:1005959203396 9426610 10.1023/a:1005959203396

[12] Saito K, Kobayashi M, Gong Z, Tanaka Y, Yamazaki M. Direct evidence for anthocyanidin synthase as a 2-oxoglutarate-dependent oxygenase: Molecular cloning and functional expression of cDNA from a red forma of Perilla frutescens. Plant J. 1999;17: 181–189. doi: 10.1046/j.1365-313X.1999.00365.x 10074715 10.1046/j.1365-313x.1999.00365.x

[13] Nakajima JI, Tanaka Y, Yamazaki M, Saito K. Reaction Mechanism from Leucoanthocyanidin to Anthocyanidin 3-Glucoside, a Key Reaction for Coloring in Anthocyanin Biosynthesis. J Biol Chem. 2001;276: 25797–25803. doi: 10.1074/jbc.M100744200 11316805 10.1074/jbc.M100744200

[14] Turnbull JJ, Nakajima JI, Welford RWD, Yamazaki M, Saito K, Schofield CJ. Mechanistic studies on three 2-oxoglutarate-dependent oxygenases of flavonoid biosynthesis: Anthocyanidin synthase, flavonol synthase, and flavanone 3*β*-hydroxylase. J Biol Chem. 2004;279: 1206–1216. doi: 10.1074/jbc.M309228200 14570878 10.1074/jbc.M309228200

[15] Sato H, Teramoto K, Masumoto Y, Tezuka N, Sakai K, Ueda S, et al “Cation-Stitching Cascade?”: Exquisite control of terpene cyclization in cyclooctatin biosynthesis. Sci Rep. Nature Publishing Group; 2015;5: 10–15. 10.1038/srep18471 PMC4683443 26681256

[16] Hong YJ, Tantillo DJ. The energetic viability of an unexpected skeletal rearrangement in cyclooctatin biosynthesis. Org Biomol Chem. Royal Society of Chemistry; 2015;13: 10273–10278. doi: 10.1039/C5OB01785H 10.1039/c5ob01785h 26371548

[17] Yu P, Patel A, Houk KN. Transannular [6 + 4] and Ambimodal Cycloaddition in the Biosynthesis of Heronamide A. J Am Chem Soc. 2015;137: 13518–13523. doi: 10.1021/jacs.5b06656 26435377 10.1021/jacs.5b06656

[18] Gaussian 09 Revision E.01 Frisch MJ, Trucks GW, Schlegel HB, Scuseria GE, Robb MA, Cheeseman JR, Scalmani G, Barone V, Mennucci B, Petersson GA, Nakatsuji H, Caricato M, Li X, Hratchian HP, Izmaylov AF, Bloino J, Zheng G, Sonnenberg JL, Hada M, Ehara M, Toyota K, Fukuda R, Hasegawa J, Ishida M, Nakajima T, Honda Y, Kitao O, Nakai H, Vreven T, Montgomery JA, Jr. Peralta JE, Ogliaro F, Bearpark M, Heyd JJ, Brothers E, Kudin KN, Staroverov VN, Kobayashi R, Normand J, Raghavachari K, Rendell A, Burant JC, Iyengar SS, Tomasi J, Cossi M, Rega N, Millam JM, Klene M, Knox JE, Cross JB, Bakken V, Adamo C, Jaramillo J, Gomperts R, Stratmann RE, Yazyev O, Austin AJ, Cammi R, Pomelli C, Ochterski JW, Martin RL, Morokuma K, Zakrzewski VG, Voth GA, Salvador P, Dannenberg JJ, Dapprich S, Daniels AD, Farkas Ö, Foresman JB, Ortiz JV, Cioslowski J, Fox DJ, Gaussian Inc. Wallingford CT 2009.

[19] Maeda, S., Osada, Y., Morokuma, K., Ohno, K. GRRM 11, Version 11.03, (2012).

[20] Maeda S, Ohno K, Morokuma K. Systematic exploration of the mechanism of chemical reactions: the global reaction route mapping (GRRM) strategy using the ADDF and AFIR methods. Phys Chem Chem Phys. 2013;15: 3683 doi: 10.1039/c3cp44063j 23389653 10.1039/c3cp44063j

[21] Ohno K, Maeda S. A scaled hypersphere search method for the topography of reaction pathways on the potential energy surface. Chem Phys Lett. 2004;384: 277–282. doi: 10.1016/j.cplett.2003.12.030

[22] Maeda S, Ohno K. Global mapping of equilibrium and transition structures on potential energy surfaces by the scaled hypersphere search method: Applications to ab initio surfaces of formaldehyde and propyne molecules. J Phys Chem A. 2005;109: 5742–5753. doi: 10.1021/jp0513162 16833907 10.1021/jp0513162

[23] Watanabe Y, Maeda S, Ohno K. Global reaction route mapping on potential energy surfaces of and. Chem Phys Lett. 2007;447: 21–26.

[24] Cossi M, Barone V. Solvent effect on vertical electronic transitions by the polarizable continuum model. J Chem Phys. 2000;112: 2427–2435. doi: 10.1063/1.480808

[25] Jacopo T, Benedetta M, Roberto C. Quantum mechanical continuum solvation models. Chem Rev. 2005;105: 2999–3093. doi: 10.1021/cr9904009 16092826 10.1021/cr9904009

[26] Plata RE, Singleton DA. A case study of the mechanism of alcohol-mediated morita baylis-hillman reactions. the importance of experimental observations. J Am Chem Soc. 2015;137: 3811–3826. doi: 10.1021/ja5111392 25714789 10.1021/ja5111392 PMC4379969

[27] Kanehisa M, Sato Y, Kawashima M, Furumichi M, Tanabe M. KEGG as a reference resource for gene and protein annotation. Nucleic Acids Res. 2016;44: D457–D462. doi: 10.1093/nar/gkv1070 26476454 10.1093/nar/gkv1070 PMC4702792

[28] Ogata H, Goto S, Sato K, Fujibuchi W, Bono H, Kanehisa M. KEGG: Kyoto encyclopedia of genes and genomes. Nucleic Acids Res. 1999;27: 29–34. doi: 10.1093/nar/27.1.29 9847135 10.1093/nar/27.1.29 PMC148090

[29] Moriya Y, Yamada T, Okuda S, Nakagawa Z, Kotera M, Tokimatsu T, et al Identification of Enzyme Genes Using Chemical Structure Alignments of Substrate-Product Pairs. J Chem Inf Model. 2016;56: 510–516. doi: 10.1021/acs.jcim.5b00216 26822930 10.1021/acs.jcim.5b00216

[30] Buchanan BB, Gruissem W, Jones RL. Biochemistry and Molecular Biology of Plants 2nd Edition Wiley, Print (2015).

