# Supplementary material for: Computational study on a puzzle in the biosynthetic pathway of anthocyanin: Why is an enzymatic oxidation/ reduction process required for a simple tautomerization?
Source: PLoS One. 2018 Jun 13;13(6):e0198944. doi: 10.1371/journal.pone.0198944 (PMC5999093; doi:10.1371/journal.pone.0198944)
Supplement: S1 File — General Methods, Energy Profiles, Cartesian Coordinates, Hypothetical Acid-Catalyzed Enzymatic Reaction (S1 Fig), Hypothetical Base-Catalyzed Enzymatic Reaction (S2 Fig), References. (DOCX) [file pone.0198944.s001.docx]

Supporting Information

Computational Study on a Puzzle in the Biosynthetic Pathway of Anthocyanin: Why is an Enzymatic Oxidation/ Reduction Process Required for a Simple Tautomerization?

Hajime Sato^a,b^ , Chao Wang^b,c^, Mami Yamazaki^a^, Kazuki Saito*^a,d^ and Masanobu Uchiyama*^b,c^

^a^ Graduate School of Pharmaceutical Sciences, Chiba University, Inohana, Chuo-ku, Chiba 260-8675, Japan

^b^ Elements Chemistry Laboratory, RIKEN, and RIKEN Center for Sustainable Resource Science (Wako campus) 2-1 Hirosawa, Wako-shi, Saitama 351-0198, Japan.

^c^ Graduate School of Pharmaceutical Sciences, The University of Tokyo, 7-3-1 Hongo, Bunkyo-ku, Tokyo 113-0033, Japan.

^d^ RIKEN Center for Sustainable Resource Science (Yokohama campus) 1-7-22 Suehiro-cho, Tsurumi-ku, Yokohama 230-0045, Japan.

*Email: ksaito@faculty.chiba-u.jp (K. S.), uchiyama@mol.f.u-tokyo.ac.jp (M. U.)

**Table of Contents**

| 1. | General Methods | S-2 |
| --- | --- | --- |
| 2. | Energy Profiles | S-3 |
| 3. | Cartesian Coordinates | S-5 |
| 3.2 | Hypothetical Acid-Catalyzed Enzymatic Reaction | S-15 |
| 3.3 | Hypothetical Base-Catalyzed Enzymatic Reaction | S-18 |
|  | References | S-20 |

**1. General Methods**

All DFT calculation were performed with Gaussian 09 program (Revision D.01)^1^ and the GRRM 11 (Version 11.03, based on Gaussian 09) program.^2^ Geometry optimizations were performed in the gas phase at the M062X level^3^ with 6-31G** basis set,^4^ without any symmetry restrictions. The vibrational frequencies were computed at the same level to check whether each optimized structure is an energy minimum (no imaginary frequency) or a transition state (one imaginary frequency) and to evaluate its zero-point vibrational energy (ZPVE) and thermal corrections at 298 K. Intrinsic reaction coordinate (IRC) were calculated to confirm the connection between the transition states and the correct reactants/products. The single-point energy considering the solvent effect of water was obtained *via* calculation of the optimized geometries at the MP2 level with 6-311++G** basis set.^5^ Solvation was evaluated by the self-consistent reaction field (SCRF) method using the polarizable continuum model (PCM).^6^ The Gibbs free energy used for discussion in this study was calculated by adding the gas-phase Gibbs free energy correction with the solution-phase single-point energy.

**2. Energy Profiles**


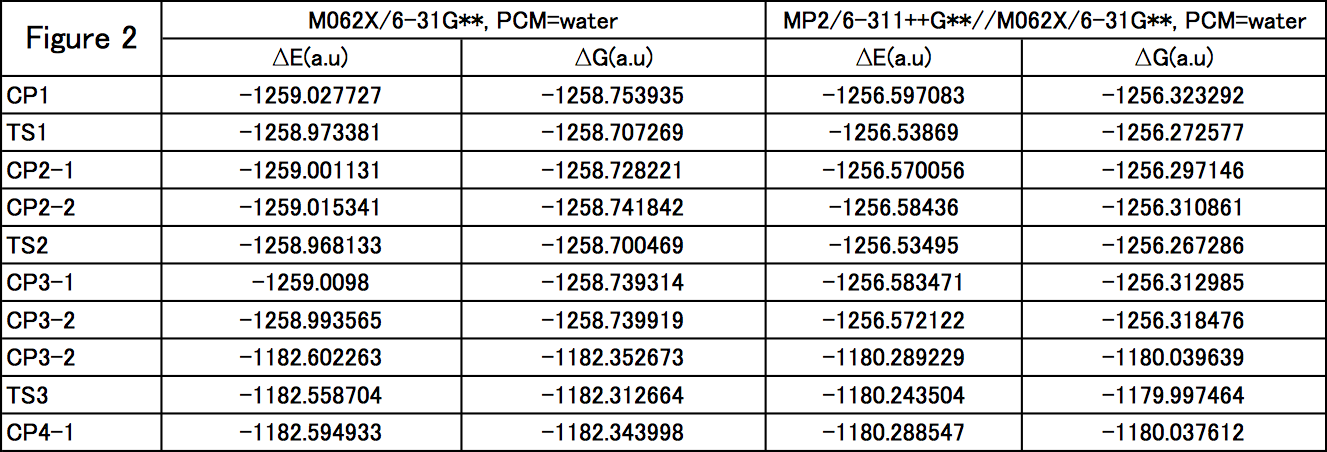


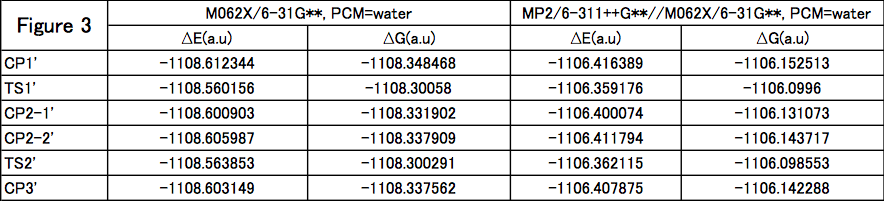


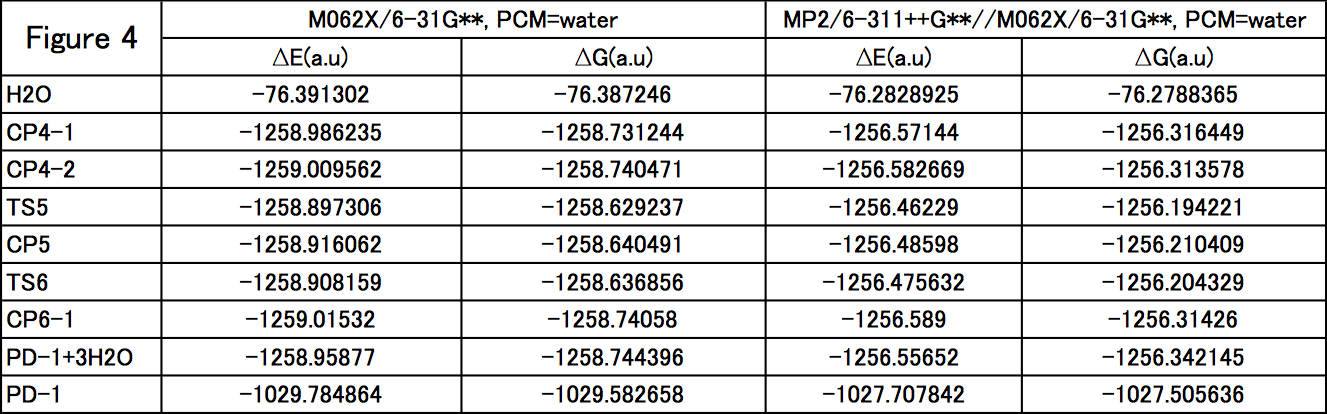


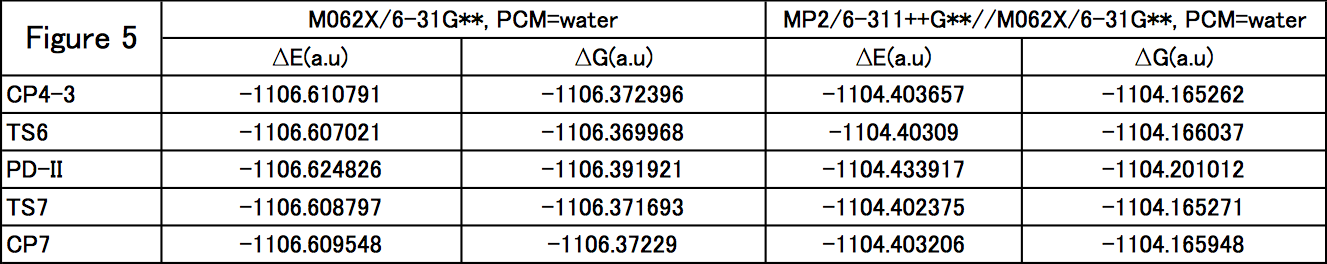


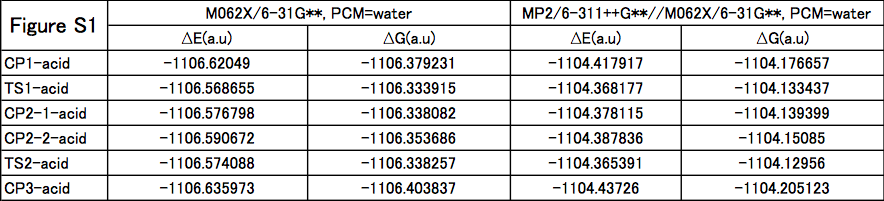


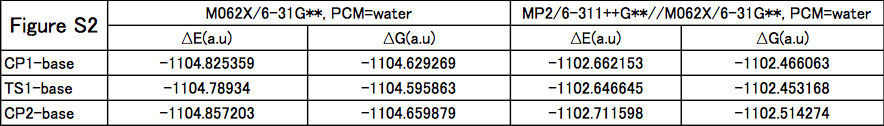


**3. Cartesian Coordinates**

**3.1 Main Route**

**CP1**

C -1.20795900 1.02597900 -0.72505600

C -2.01832000 -0.14846800 -0.47027500

C -1.41487800 -1.37313800 -0.11469600

C -3.42790000 -0.04794900 -0.42122100

C -2.17996100 -2.46755400 0.25640800

C -3.56902800 -2.33149800 0.28986800

C -4.20593800 -1.13152500 -0.04284400

H -1.71252200 -3.40973200 0.50980400

H -5.28533600 -1.02808400 -0.01490200

O -0.07395100 -1.52609300 -0.10586900

O 1.01286000 1.83296000 -1.08903000

H 1.66602400 2.17616300 -0.43925700

C 0.71383500 -0.54572700 -0.79188600

C 0.26706900 0.87784600 -0.40854900

C 2.16108400 -0.78241400 -0.45321100

C 3.15758600 -0.32433900 -1.31920500

C 2.53337600 -1.39079700 0.74259200

C 4.49942900 -0.47238000 -1.00228600

H 2.87308700 0.16448400 -2.24596700

C 3.87672500 -1.55139500 1.06636600

H 1.76729100 -1.75250600 1.41957400

C 4.86191700 -1.09010800 0.19551300

H 5.28162000 -0.12309400 -1.66682800

H 4.15920400 -2.03428400 1.99869600

H 0.55446100 -0.66482200 -1.87425800

O 6.19132700 -1.21728200 0.45656700

H 6.30786000 -1.65531400 1.30688200

O -1.68343500 2.09453900 -1.11782700

H -3.33463700 1.72186700 -1.06589900

H 1.25768200 3.26585700 1.17896800

H 2.80908600 3.43824500 1.16361000

H 0.35857600 0.98173400 0.68671000

O 2.11315400 2.77512800 1.14008300

O -4.27255400 -3.42464300 0.65549600

H -5.21644700 -3.23092400 0.62132800

O -4.03126500 1.12554800 -0.69285500

O -0.30966100 3.81048300 0.71971500

H -1.07294800 3.39375600 1.15464000

H -0.38562400 3.51476100 -0.20104500

O -2.47742900 2.22387500 1.66799300

H -2.85375800 2.08238100 2.54162100

H -3.20820800 2.12418900 1.04303700

**TS1**

C -1.18037700 0.94223500 -0.62691800

C -2.01948500 -0.22306000 -0.32800100

C -1.42626000 -1.46470300 -0.03622100

C -3.42623900 -0.12762200 -0.28818900

C -2.17897500 -2.57310400 0.30861200

C -3.57017400 -2.44167700 0.34851600

C -4.20074100 -1.23808100 0.04514700

H -1.70558100 -3.52012900 0.53109600

H -5.28079500 -1.13628600 0.06462500

O -0.07045600 -1.60845200 -0.03252900

O 0.97208800 1.69321800 -1.30378600

H 1.89829000 1.55592100 -1.06011200

C 0.67044000 -0.71553400 -0.86405900

C 0.20741100 0.70907600 -0.62478900

C 2.13159100 -0.85655400 -0.50140800

C 3.12529100 -0.60434100 -1.45052000

C 2.51380700 -1.13484500 0.81226100

C 4.47125400 -0.62443500 -1.10341300

H 2.83948400 -0.39470900 -2.47848300

C 3.85794800 -1.16621200 1.16961600

H 1.74544900 -1.34939700 1.54911100

C 4.84069200 -0.90879000 0.21116800

H 5.24926300 -0.43556500 -1.83449000

H 4.14648400 -1.40273800 2.19115400

H 0.52700100 -1.01244600 -1.91832200

O 6.16835000 -0.92169900 0.49847800

H 6.29757500 -1.16914300 1.42090900

O -1.69855200 2.09722200 -0.80270800

H -3.32757400 1.66724300 -0.79926200

H 0.75923000 2.74411100 1.21965200

H 1.82272900 1.39034800 1.88189100

H 0.51749000 1.05554400 0.88453800

O 0.90310800 1.64876800 1.73537600

O -4.27407400 -3.55109800 0.68357800

H -5.21582900 -3.34656300 0.66515700

O -4.04570500 1.02640900 -0.57110900

O 0.48188900 3.67087200 0.52481800

H -0.46326800 3.96363600 0.75007600

H 0.43286700 3.25168400 -0.36421400

O -2.00171800 4.03404800 1.00062100

H -2.45614500 4.75514000 0.55213000

H -2.16320600 3.23491600 0.45182900

**CP2-1**

C -1.19304900 1.04653000 -0.75873600

C -1.99044000 -0.15762600 -0.49900600

C -1.35609700 -1.23981000 0.13055600

C -3.37379600 -0.24982900 -0.72319100

C -2.03445800 -2.36287900 0.56257200

C -3.41346800 -2.41459000 0.33870000

C -4.08081200 -1.37805700 -0.30726700

H -1.51830700 -3.18525300 1.04010700

H -5.14882000 -1.41896600 -0.49559200

O 0.00126400 -1.16073600 0.35659900

O 0.97799700 1.99783900 -0.96491900

H 1.66208900 1.90523600 -0.28005500

C 0.68915200 -0.49519600 -0.71177100

C 0.14128000 0.92466400 -0.81167000

C 2.17636100 -0.60465400 -0.46357600

C 3.07809900 0.00015000 -1.34759200

C 2.68283200 -1.33320400 0.61213900

C 4.44757100 -0.09647200 -1.14859500

H 2.70092400 0.56038100 -2.19773500

C 4.05636500 -1.43179400 0.82272000

H 1.99646900 -1.83697100 1.28413100

C 4.94278900 -0.81138700 -0.05653400

H 5.15141400 0.37085100 -1.82801700

H 4.43775300 -2.00578900 1.66376300

H 0.44511200 -1.03843300 -1.63944900

O 6.29126500 -0.86883700 0.09274700

H 6.50958000 -1.41165900 0.85859500

O -1.83438300 2.24728800 -0.91444500

H -3.45712300 1.46324000 -1.52794800

H 0.49388800 2.94909500 1.63955400

H 2.15446200 0.79765700 2.05759300

H 0.70064300 0.53764100 1.63557200

O 1.33734700 1.24743100 1.81452800

O -4.05847900 -3.53193600 0.76156000

H -4.99347300 -3.46243200 0.53837600

O -4.06632700 0.72742600 -1.34344800

O -0.10087400 3.59429500 1.22468600

H -1.70219700 3.20496500 1.59742600

H -0.03008800 3.40060600 0.27590800

O -2.61502400 2.84004100 1.47306900

H -3.22288900 3.57841000 1.58048300

H -2.27569200 2.47384900 -0.03821600

**CP2-2**

C 0.92103300 1.26658900 0.32236900

C 1.93847000 0.23073500 0.12265500

C 1.79200500 -0.98041500 0.82111000

C 3.06156900 0.36463500 -0.71394400

C 2.69528800 -2.02364000 0.70254900

C 3.79642200 -1.85678500 -0.13725100

C 3.98269800 -0.67559800 -0.84532600

H 2.54833600 -2.94297600 1.25379500

H 4.83556400 -0.52680600 -1.49949700

O 0.75679900 -1.17904700 1.68770200

O -1.19050400 1.85428300 1.35124200

H 0.34270400 2.69615000 -0.86141200

C -0.40737700 -0.36725900 1.62993600

C -0.15390800 1.01355700 1.08076700

C -1.51624000 -1.03020900 0.83339400

C -1.26664300 -1.50830500 -0.45887400

C -2.81506900 -1.08384200 1.32937100

C -2.29387500 -2.02856400 -1.23454600

H -0.25733600 -1.46819100 -0.86074000

C -3.85772200 -1.59575400 0.55917500

H -3.02252200 -0.70668000 2.32707200

C -3.59754200 -2.07305300 -0.72392000

H -2.11435600 -2.41286900 -2.23262200

H -4.86911500 -1.62462100 0.95594200

H -0.74380300 -0.25405100 2.66707300

O -4.56014500 -2.58929600 -1.52947200

H -5.40957800 -2.56655800 -1.07439900

O 1.12611000 2.49757600 -0.28458800

H 2.63766100 2.14563300 -1.18246600

H -1.79059900 2.47665200 -1.39242300

H -2.56950100 1.43411100 0.10049700

H -2.72595800 0.48671700 -1.10022100

O -2.84702700 1.40898800 -0.83418900

O 4.66334100 -2.89925600 -0.23016500

H 5.37565400 -2.66321400 -0.83469100

O 3.31032100 1.48630400 -1.42535000

O -1.08452000 3.16831500 -1.56609200

H -1.10703400 3.37319100 -2.50602000

H -0.92458100 4.37883200 -0.01937500

O -0.58833600 4.39689700 0.89269800

H 0.36732600 4.29029300 0.78478500

H -0.93582700 2.81371300 1.25561600

**TS2**

C 0.53339100 1.36024200 0.27111100

C 1.70449800 0.46761400 0.11378600

C 1.81799900 -0.66358800 0.93342100

C 2.71202800 0.65846100 -0.84725900

C 2.85526500 -1.57822100 0.81623000

C 3.83093000 -1.35841800 -0.15314000

C 3.76697800 -0.24343500 -0.98114300

H 2.90427200 -2.43740100 1.47295600

H 4.52523500 -0.04017200 -1.73053200

O 0.90870500 -0.91621300 1.92835500

O -1.30941100 1.84686300 1.72028200

H 0.00746400 2.89513800 -0.83307200

C -0.37364200 -0.32447800 1.85003800

C -0.36906800 1.10824500 1.30862100

C -1.33765200 -1.15025700 1.01223200

C -0.89040800 -2.19402300 0.19798900

C -2.68726600 -0.78827000 0.96761400

C -1.76537800 -2.85206300 -0.65910600

H 0.15340100 -2.48811900 0.22727400

C -3.56937800 -1.43794700 0.10581500

H -3.03505000 0.03375100 1.58634700

C -3.10839100 -2.47749600 -0.70911100

H -1.42761300 -3.66071500 -1.29739700

H -4.61801300 -1.15098500 0.08024300

H -0.73687800 -0.25617300 2.87922400

O -3.91815000 -3.15238700 -1.56559000

H -4.82643800 -2.84724600 -1.46196800

O 0.69372800 2.69820200 -0.17539900

H 2.09300500 2.37940800 -1.30221400

H -1.70920800 1.64948200 -1.59967800

H -2.01652600 0.03238300 -1.17746900

H -0.56167600 0.75231100 -0.74458000

O -1.34370200 0.64777700 -1.51976900

O 4.83487600 -2.27345400 -0.24410400

H 5.43908200 -2.00535700 -0.94503500

O 2.70943600 1.72921700 -1.67920900

O -1.81272100 3.02642600 -1.55838200

H -2.19346100 3.52492200 -2.28691500

H -1.90608600 3.54200100 -0.61634000

O -1.79369700 4.04013600 0.65388400

H -0.99880900 4.58095700 0.73373100

H -1.56465200 3.16510300 1.17229100

**CP3-1**

C 0.88860100 0.88281600 -0.49499200

C 2.04822000 -0.06013800 -0.26410300

C 1.70492300 -1.38855200 0.00833900

C 3.40777300 0.26152700 -0.36522800

C 2.64102200 -2.39155300 0.18123100

C 3.98931000 -2.04335800 0.07380000

C 4.37375800 -0.73277000 -0.18855100

H 2.33973400 -3.40944400 0.39025700

H 5.41881800 -0.45271700 -0.27507800

O 0.36849900 -1.71920600 0.05119700

O -0.50764200 1.45215300 1.40531900

H 0.57493800 2.77204500 -0.90797000

C -0.43955300 -0.86287700 0.84080600

C -0.08019600 0.61171800 0.64373900

C -1.89552600 -1.08550400 0.49704700

C -2.26875900 -1.74838500 -0.67265600

C -2.88269500 -0.55199500 1.32434700

C -3.60874700 -1.88249700 -1.00938000

H -1.50049700 -2.15309200 -1.32076900

C -4.22768400 -0.67312000 0.98832900

H -2.59830500 -0.01859300 2.22635200

C -4.59327700 -1.34445700 -0.17988200

H -3.91447600 -2.39431700 -1.91478000

H -4.99215800 -0.25520600 1.63847800

H -0.26726200 -1.07376700 1.90638000

O -5.88816500 -1.50367800 -0.55972600

H -6.46836000 -1.09981600 0.09516500

O 1.29535400 2.22991400 -0.52073200

H 3.07432100 2.08368400 -0.73928800

H -1.81771800 2.30627500 -1.18387600

H -2.61714300 1.03445900 -0.81023300

H 0.35230200 0.60944100 -1.41517200

O -1.91517600 1.36225800 -1.38396500

O 4.89611500 -3.03936800 0.24708100

H 5.78521500 -2.67992300 0.15203000

O 3.85047800 1.50442500 -0.64676100

O -0.79490700 3.90198100 -0.95894100

H -0.82356900 4.67097100 -1.53573200

H -0.53774700 4.20746300 -0.05217900

O 0.26029500 4.14570700 1.44516000

H 1.17875400 3.99437600 1.18842600

H -0.04866100 3.23955100 1.63186800

**CP4-1**

C -2.18170600 -0.29034000 -0.45432700

C -1.57450600 0.94681100 -0.26767700

C -3.58033900 -0.37051300 -0.31766700

C -2.31482400 2.09606600 0.00201900

C -3.69864700 1.98744500 0.09576500

C -4.34226200 0.75734000 -0.05334400

H -1.79895000 3.04149600 0.13052500

H -5.41727900 0.68316100 0.04741500

O -0.22676200 1.11426600 -0.36491700

O -4.21826200 -1.55681400 -0.46246000

O -4.48745100 3.06197500 0.35383300

H -3.93767900 3.84888800 0.43781200

C 0.58322600 0.01165700 -0.53597300

C 0.08421800 -1.21846800 -0.73366900

C 2.00980300 0.35937700 -0.41076300

C 2.39883300 1.41783400 0.42243200

C 3.00256900 -0.35206700 -1.09550100

C 3.73666000 1.74723400 0.58308400

H 1.63557900 1.98154700 0.94724800

C 4.34402600 -0.02767800 -0.93788700

H 2.71900200 -1.16919100 -1.74945300

C 4.71650000 1.02281200 -0.09750800

H 4.04553500 2.56155700 1.22883500

H 5.10437800 -0.58821100 -1.47589300

H -1.00251300 -1.71101800 1.69670800

O 6.01095500 1.38351600 0.09797800

H 6.58581200 0.82124800 -0.43334800

C -1.38281900 -1.49826300 -0.81324000

H -1.65024400 -1.88043300 -1.81113200

O -1.69536500 -2.55601100 0.13010500

H -3.58219500 -2.27295800 -0.30790500

O 0.82787100 -2.36240000 -0.79744600

H -1.04732800 -3.25452500 -0.05708600

O -0.40131100 -1.31108900 2.35018700

H -0.79684700 -1.47298200 3.21208100

H 2.61292900 -1.30796700 1.43126400

O 2.06908600 -2.10299400 1.49790500

H 1.44544500 -2.36296800 -0.01117000

H 1.24987900 -1.81671100 1.95815100

**TS3**

C -2.22795700 -0.36461100 -0.49658100

C -1.59990400 0.87989400 -0.53899400

C -3.56879400 -0.41729100 -0.07498200

C -2.25035400 2.06131700 -0.20547300

C -3.58661800 1.97989000 0.18807100

C -4.24039300 0.75357600 0.27264100

H -1.71253300 3.00250200 -0.24730800

H -5.27410900 0.70600700 0.59086100

O -0.28743200 0.94477800 -0.92762500

O -4.25658300 -1.57442700 0.00206900

O -4.30080700 3.08341900 0.53442200

H -3.75614700 3.86773200 0.40627500

C 0.52115300 -0.11809500 -0.44996300

C 0.00589600 -1.41930700 -0.56052500

C 1.95981000 0.22834900 -0.41016800

C 2.34631500 1.54442500 -0.11724400

C 2.96456000 -0.72583800 -0.61174000

C 3.68314100 1.89824700 -0.02571500

H 1.57692200 2.29564200 0.02584300

C 4.30983300 -0.38033400 -0.50411800

H 2.69081500 -1.74476400 -0.86728900

C 4.67445900 0.93341300 -0.21198200

H 3.98392700 2.91679200 0.19323800

H 5.07711600 -1.13239200 -0.67309500

H 0.25214500 -0.14970500 1.03500800

O 5.96945700 1.33269200 -0.09704800

H 6.55316100 0.59023900 -0.28845700

C -1.44249100 -1.53608200 -1.03979700

H -1.42666800 -1.48907000 -2.14168700

O -1.96038100 -2.78146100 -0.63181100

H -3.64448800 -2.30910200 -0.17863200

O 0.54767300 -2.48947000 -0.15235300

H -1.18681700 -3.28137100 -0.30987500

O 0.27275400 -0.19942500 2.18525300

H -0.60542300 -0.45705000 2.50027400

H 2.74225100 -1.64602000 1.78519700

O 1.88552500 -2.03046500 2.01830800

H 1.49835400 -2.35865200 1.15011200

H 0.97789600 -1.00122600 2.28923200

**CP3-2**

C -2.24738100 -0.35419800 -0.55726800

C -1.75696600 0.91699800 -0.26098200

C -3.62249900 -0.59355300 -0.41136000

C -2.57226800 1.95779700 0.15146600

C -3.94070400 1.69943500 0.27168000

C -4.46343900 0.43782000 0.00519900

H -2.13875600 2.92630300 0.37542600

H -5.52470300 0.25307700 0.11342300

O -0.40744300 1.14028100 -0.42403300

O -4.18685300 -1.78945300 -0.67353700

O -4.81753800 2.65715900 0.66872100

H -4.34289200 3.48183800 0.82034000

C 0.41812600 0.16584900 0.22072900

C -0.01806600 -1.23549700 -0.17617700

C 1.85718600 0.42452600 -0.14311900

C 2.78423500 0.78970200 0.83635000

C 2.27603700 0.31300200 -1.46880100

C 4.11421500 1.01279200 0.49939700

H 2.45526700 0.89063600 1.86762100

C 3.60054900 0.54242300 -1.81763200

H 1.55765300 0.05386500 -2.24253800

C 4.52471900 0.88878100 -0.82949500

H 4.84707400 1.30018800 1.24525100

H 3.91873800 0.45384900 -2.85312400

H 0.29751100 0.22230700 1.31399800

O 5.83366000 1.11896000 -1.10062700

H 5.99271700 1.01840700 -2.04592400

C -1.22616900 -1.33150000 -1.09399100

H -0.89427400 -0.99922000 -2.09356200

O -1.67596700 -2.66054800 -1.16627500

H -3.48239200 -2.42040700 -0.89672400

O 0.50266000 -2.25190800 0.24079000

H -1.04969700 -3.19436600 -0.64879700

O 1.08906000 -0.20290500 3.30102700

H 0.69896900 -0.45220300 4.14330100

H 3.35881400 -1.55284000 1.63649800

O 2.69073700 -2.08360400 2.08651500

H 2.03038400 -2.27489100 1.40049600

H 1.68360100 -0.93664800 3.04770400

**CP1'**

C 1.76939600 0.38490300 0.37651600

C 2.28722900 -0.97955500 0.17423200

C 1.39307400 -2.02938200 -0.08154600

C 3.66617000 -1.23125600 0.17734200

C 1.87918600 -3.31135000 -0.35368000

C 3.24633300 -3.53465600 -0.35673600

C 4.15029200 -2.49900800 -0.08450500

H 1.16713500 -4.10531500 -0.54725800

H 5.21694300 -2.69156100 -0.08549400

O 0.05047900 -1.86109200 -0.08993100

O -0.20154500 1.65650400 0.87453600

H -0.72111200 2.19427200 0.23202700

C -0.45856000 -0.72531200 0.61621300

C 0.26920900 0.55414200 0.16772200

C -1.94157100 -0.64065500 0.37230200

C -2.75414800 0.05181900 1.27444900

C -2.51787500 -1.19012800 -0.77011600

C -4.11436100 0.19201000 1.04458400

H -2.30844700 0.49460500 2.15974100

C -3.88298200 -1.06108700 -1.00662100

H -1.89691100 -1.73329400 -1.47392500

C -4.68321700 -0.36803500 -0.10047900

H -4.75520200 0.72463200 1.73817500

H -4.32590400 -1.49940300 -1.89760500

H -0.25885800 -0.86320900 1.69022100

O -6.02268800 -0.20760500 -0.27554500

H -6.29087700 -0.63759500 -1.09514500

O 2.48481800 1.33343400 0.65297300

H -0.07107600 3.54235800 -1.03060500

H -1.60692200 3.75526500 -1.21097600

H 0.11303700 0.66795500 -0.91933900

O -0.92309700 3.07908900 -1.22419700

O 1.33055500 3.92252700 -0.12803900

H 2.22230100 3.84782100 -0.51039400

H 1.28865000 3.18757800 0.50522900

O 4.05290100 3.32857800 -0.56779100

H 4.42639100 3.87130500 0.13451600

H 3.82890800 2.48997700 -0.13759600

H 4.33262700 -0.39911400 0.38031600

H 3.61887800 -4.53245000 -0.56600700

**TS1'**

C 1.65863500 0.56879500 0.66657000

C 2.32229200 -0.70058900 0.26959600

C 1.54703900 -1.82367400 -0.04693600

C 3.71209500 -0.79035800 0.19645200

C 2.15448700 -3.01056500 -0.45003700

C 3.54083100 -3.07808600 -0.52311800

C 4.32671400 -1.97216000 -0.19479000

H 1.52606300 -3.85997000 -0.69349300

H 5.40797600 -2.03593200 -0.24786500

O 0.18356700 -1.79587600 -0.02135500

O -0.37110400 1.57280100 1.37676300

H -1.29527500 1.61080000 1.09500400

C -0.42266400 -0.83333500 0.84209700

C 0.24863300 0.51569700 0.66691500

C -1.88427200 -0.73544700 0.46590300

C -2.84171600 -0.38994800 1.42266400

C -2.28576300 -0.87905400 -0.86325200

C -4.17126200 -0.19156800 1.06873700

H -2.54013800 -0.27811100 2.46122000

C -3.61511500 -0.69208400 -1.22825300

H -1.54700000 -1.15992400 -1.60799000

C -4.56146400 -0.34620200 -0.26134400

H -4.92201400 0.07176100 1.80531000

H -3.92198200 -0.82411200 -2.26325500

H -0.34152500 -1.19275500 1.88354800

O -5.87333300 -0.14736100 -0.55553500

H -6.02382200 -0.31948100 -1.49171800

O 2.32295800 1.61258100 0.88950200

H 0.17368300 2.70093400 -1.08483200

H -1.14204900 1.63873000 -1.81707400

H 0.04251700 0.97989200 -0.81730800

O -0.19174200 1.68196800 -1.64933800

O 0.62557800 3.52852100 -0.35901600

H 1.61187900 3.64249900 -0.57287100

H 0.61128700 3.06973100 0.51043900

O 3.16254400 3.45906800 -0.74702100

H 3.68061500 4.09197200 -0.23856000

H 3.14238900 2.63450200 -0.20303400

H 4.28676700 0.09333600 0.45464100

H 4.01205500 -4.00603600 -0.83137600

**CP2-1'**

C 1.51609300 1.01362000 0.44526000

C 2.43171700 -0.05453100 0.03645000

C 2.16354600 -1.36169600 0.46526200

C 3.55624300 0.17965000 -0.75380800

C 2.99959100 -2.41172600 0.10352200

C 4.11639600 -2.15992000 -0.68933000

C 4.39832500 -0.86595500 -1.11983400

H 2.76078800 -3.40887700 0.45629500

H 5.27184300 -0.67088500 -1.73226000

O 1.10116300 -1.66666400 1.26473600

O -0.44914000 1.64413900 1.63181400

H -1.22905400 1.62900100 1.02232700

C 0.07864700 -0.71156900 1.53032400

C 0.44241400 0.71324200 1.18173100

C -1.19372200 -1.07055300 0.78269500

C -2.44114400 -0.97099900 1.39625000

C -1.13340700 -1.38775900 -0.57495000

C -3.61397600 -1.18704700 0.67818700

H -2.49867200 -0.71034400 2.44961500

C -2.29474800 -1.61711700 -1.30359800

H -0.16649900 -1.45323200 -1.06727200

C -3.54124300 -1.52195800 -0.67530800

H -4.58944200 -1.11755100 1.14638400

H -2.23848000 -1.87478200 -2.35842900

H -0.12178400 -0.75932900 2.60836900

O -4.71123200 -1.74020900 -1.33041300

H -4.52890400 -2.00339000 -2.23950900

O 1.79926100 2.30187200 0.06697000

H -1.45893500 3.86236000 0.50887900

H -2.72972700 0.97371500 -0.44952300

H -1.58740900 1.83485500 -1.08041100

O -2.22869300 1.79417800 -0.34602000

O -0.56191200 4.13535000 0.27504900

H -0.30812100 3.42758700 -1.19171600

H 0.00719800 3.56201200 0.82032200

O -0.11358900 2.70946100 -1.85544900

H -0.09123100 3.11587700 -2.72719400

H 1.38394500 2.43627400 -0.80757000

H 3.76156700 1.19958700 -1.06103700

H 4.76766100 -2.98199300 -0.96788500

**CP2-2'**

C 1.74360600 0.77239200 0.37503500

C 1.91298600 -0.63365100 0.00709500

C 1.33902400 -1.61922800 0.82779700

C 2.64729200 -1.04541500 -1.10975600

C 1.49769600 -2.96962200 0.54805100

C 2.23037800 -3.35941500 -0.57168800

C 2.79956900 -2.39871000 -1.40342000

H 1.05165100 -3.69581700 1.21973200

H 3.37198400 -2.70071200 -2.27374400

O 0.65475700 -1.26136800 1.95735700

O 0.48271100 2.29279200 1.77011200

H 2.06286800 1.92188700 -1.10041100

C -0.03500800 -0.02056400 1.88919400

C 0.82357000 1.08288400 1.30581500

C -1.33494100 -0.16412100 1.09878800

C -1.83176200 -1.42749300 0.76745000

C -2.04384100 0.96356700 0.67426400

C -2.99366500 -1.56875700 0.01480000

H -1.29673200 -2.31122800 1.09637700

C -3.21130500 0.83314700 -0.07174900

H -1.67257200 1.95236200 0.92216000

C -3.69287000 -0.43601600 -0.40337200

H -3.37845400 -2.54658500 -0.25241700

H -3.75041700 1.71989200 -0.39598900

H -0.25546000 0.24311500 2.92820000

O -4.82507500 -0.62804000 -1.12856800

H -5.22566400 0.22427900 -1.33349500

O 2.52879400 1.71038900 -0.26517600

H 0.10747100 1.48027700 -2.00642600

H -1.31105700 -0.20730600 -1.52541600

H 0.14721700 -0.67892700 -1.62095400

O -0.49774600 -0.08679800 -2.03524600

O 0.61591700 2.32872900 -2.08250800

H 0.69214800 2.50076600 -3.02637800

H 0.79026900 3.73272900 -0.76874300

O 1.18726000 4.15822300 0.01175900

H 2.11240400 3.88030300 -0.03148200

H 0.80964900 3.02067500 1.17728800

H 3.11675900 -0.28657200 -1.72724800

H 2.35061900 -4.41454500 -0.79272500

**TS2'**

C 1.25786900 0.97567300 0.09065300

C 2.09477600 -0.21246500 -0.15993000

C 1.92778900 -1.35640000 0.63248200

C 3.05381000 -0.25140500 -1.17335500

C 2.68371100 -2.50145800 0.41475200

C 3.63065400 -2.52086900 -0.60606400

C 3.81801300 -1.39258100 -1.39883000

H 2.52433800 -3.35559200 1.06449100

H 4.56088700 -1.39685800 -2.18935100

O 1.05208500 -1.38428500 1.68867600

O -0.20536800 1.94611600 1.71620100

H 1.37038800 2.42209300 -1.22050000

C 0.02718900 -0.40976300 1.75349100

C 0.42120600 0.97633800 1.22103600

C -1.22480500 -0.85497700 1.01171800

C -1.20990600 -1.95618600 0.15218300

C -2.38861300 -0.08605400 1.10363600

C -2.32459400 -2.27645800 -0.61446700

H -0.31393300 -2.56252400 0.07501500

C -3.50803600 -0.39580200 0.33365900

H -2.39846400 0.78149400 1.75663900

C -3.47877000 -1.49788200 -0.52701800

H -2.32154200 -3.12693600 -1.28686900

H -4.40957200 0.20701300 0.41463200

H -0.19558300 -0.27427400 2.81557300

O -4.53801000 -1.85431300 -1.29950900

H -5.28750100 -1.27997400 -1.10669600

O 1.76490000 2.20659600 -0.36410500

H -0.89506400 2.01351800 -1.59201100

H -1.66440300 0.56568500 -1.11410300

H -0.01613300 0.77046000 -0.79153100

O -0.86074800 0.93703300 -1.51683000

O -0.58951800 3.33890800 -1.62162500

H -1.12033800 3.93678700 -2.15601900

H -0.36821600 3.78010600 -0.67984500

O 0.05887000 4.21578600 0.58861300

H 1.02092200 4.25535800 0.49974100

H -0.05499700 3.35197500 1.11905300

H 3.20862800 0.64196900 -1.76937900

H 4.22113300 -3.41531700 -0.77388700

**CP3'**

C 1.32908800 0.57008900 -0.60965200

C 2.37686700 -0.48603300 -0.36667800

C 1.89929200 -1.73675700 0.02957500

C 3.74328800 -0.29083100 -0.52174500

C 2.76211200 -2.80285400 0.24146400

C 4.12941300 -2.60266100 0.06419900

C 4.62025600 -1.35262300 -0.30724800

H 2.35455500 -3.76210200 0.54033500

H 5.68728300 -1.20578700 -0.43524700

O 0.54066100 -1.91999400 0.15946700

O 0.14449000 1.42230900 1.32895200

H 1.19460300 2.44360500 -1.08735600

C -0.13889000 -0.90767400 0.88920900

C 0.41095200 0.49443000 0.59864900

C -1.61127000 -0.94124500 0.54550500

C -2.07327700 -1.58931200 -0.60044900

C -2.51371500 -0.23793500 1.34244000

C -3.41785100 -1.54317400 -0.94281300

H -1.37008900 -2.12761900 -1.22506700

C -3.86126500 -0.17850600 1.00087000

H -2.15657500 0.28707500 2.22340000

C -4.31681500 -0.83682400 -0.14319800

H -3.79186000 -2.04122800 -1.83006300

H -4.55924100 0.37120800 1.62727800

H 0.00333600 -1.07388600 1.96636300

O -5.62016700 -0.82447300 -0.52631200

H -6.13689200 -0.31014000 0.10381900

O 1.89796000 1.83129300 -0.79168900

H -1.14738300 2.43565700 -1.10998300

H -2.07743100 1.24637500 -0.78326600

H 0.70321200 0.27546600 -1.46629600

O -1.37613200 1.53205400 -1.37995600

O 0.05143100 3.88288800 -0.85768500

H 0.14835200 4.65886900 -1.41740600

H 0.54269600 4.06229500 -0.01996800

O 1.66130500 3.79732500 1.27638000

H 2.18757900 3.16686800 0.75761900

H 1.07770000 3.19366900 1.75896200

H 4.09983000 0.68788200 -0.82360600

H 4.81483900 -3.42795900 0.22646600

**CP4-2**

C 2.19124800 0.08537800 -0.34989400

C 1.57248100 -1.14227300 -0.15225700

C 3.59543700 0.10610000 -0.38696900

C 2.29691200 -2.32567200 0.01713900

C 3.68146600 -2.26352400 -0.01563200

C 4.34433300 -1.04944000 -0.22388600

H 1.76255200 -3.25887300 0.15844800

H 5.42880800 -1.03754100 -0.25083200

O 0.22345600 -1.27270300 -0.15109400

O 4.16837400 1.31663100 -0.59979500

H 5.12647000 1.21722100 -0.61984100

O 4.46348100 -3.36343100 0.13962500

H 3.90196200 -4.13453800 0.27519900

C -0.56695000 -0.17890700 -0.43712100

C -0.05377000 1.05141000 -0.61042300

C -1.99627000 -0.53224900 -0.42451200

C -2.94678700 0.14358200 -1.20532500

C -2.43804300 -1.55345500 0.42559800

C -4.29391500 -0.17927600 -1.12489800

H -2.62178900 0.93070000 -1.87480700

C -3.78668000 -1.88021500 0.51038100

H -1.71240300 -2.08361500 1.03230100

C -4.72085600 -1.19285400 -0.26531900

H -5.03306100 0.33685400 -1.72727900

H -4.11427600 -2.67123900 1.18070800

H -0.77225200 1.02507700 2.22302100

O -6.05192300 -1.46359200 -0.22589100

H -6.21331000 -2.19037300 0.38579000

C 1.40626900 1.35856400 -0.42620700

H 1.76698600 1.98335700 -1.25336100

H -2.87071300 1.02514900 1.42717800

O 0.12363400 0.70434500 2.47296100

H -1.96076700 2.03275700 0.73634500

O -2.25827400 1.74630300 1.61951600

H 0.19239300 0.82653700 3.42471800

O -0.88898400 2.11193000 -0.83150100

O 1.59395000 2.16800000 0.74083800

H 1.17935200 1.67630400 1.48786200

H 1.08933100 3.75336900 0.19346400

O 0.55481500 4.29832300 -0.42662600

H 0.09523600 4.95321400 0.10786300

H -0.38200700 2.96617700 -0.79509400

**TS5**

C 2.03770500 0.04051600 -0.22524400

C 1.48362600 -0.95605600 0.57012200

C 3.28956900 -0.21801700 -0.80609800

C 2.14376900 -2.15967600 0.82600600

C 3.38987800 -2.37013100 0.25198300

C 3.97084300 -1.40616600 -0.57411200

H 1.65944400 -2.90447600 1.44812500

H 4.93879000 -1.60608500 -1.02162400

O 0.25543400 -0.84041200 1.14520100

O 3.79228100 0.74864700 -1.61395300

H 4.64059900 0.45673100 -1.96516400

O 4.09603500 -3.51464100 0.44867900

H 3.58798800 -4.10446900 1.01636600

C -0.60308600 0.20934100 0.79363900

C 0.01787800 1.34120300 0.19535000

C -1.88512600 -0.35591600 0.21208400

C -2.09017700 -0.27823300 -1.16781500

C -2.79882000 -1.06932200 0.98873600

C -3.19092000 -0.87902400 -1.76170500

H -1.37814600 0.26935000 -1.77645900

C -3.90296000 -1.67744900 0.40388400

H -2.63775200 -1.14529300 2.05765700

C -4.10274500 -1.58039500 -0.97345100

H -3.36260700 -0.81923300 -2.83050800

H -4.61140700 -2.22999100 1.01671300

H -1.96337700 1.60741100 1.97177400

O -5.16691500 -2.15038300 -1.60007400

H -5.71461300 -2.60072700 -0.94784200

C 1.34411100 1.35464600 -0.42238500

H 1.31335300 1.61290300 -1.49216800

H -2.39076100 3.26115300 1.93154000

O -1.15248900 0.61971900 2.32926700

H -1.77525500 2.44882700 0.44945900

O -2.46785400 2.44413800 1.42767400

H -0.34599500 1.02607300 2.67824900

O -0.94718700 2.26336400 -0.40318800

O 2.20928200 2.44213600 0.10202400

H 2.08972700 2.38085800 1.06031300

H 1.38952600 3.76490800 -0.51161200

O 0.60942600 4.28721400 -0.83896200

H 0.56999000 5.09575500 -0.32068600

H -0.45488500 3.11615000 -0.60344600

**CP5**

C -1.79686300 0.21282300 0.12409200

C -1.40892600 -0.57898500 -0.95283000

C -2.89833900 -0.21261800 0.88011000

C -2.09431100 -1.74287800 -1.30338700

C -3.19699800 -2.11951100 -0.54705200

C -3.60331600 -1.36576000 0.55508200

H -1.74245900 -2.32999300 -2.14484100

H -4.45667500 -1.69702600 1.13761600

O -0.34482000 -0.24293200 -1.72692100

O -3.23324600 0.55444300 1.94849700

H -3.99742600 0.16643000 2.38851800

O -3.91882600 -3.23794400 -0.82385200

H -3.53702500 -3.67492400 -1.59290500

C 0.70625400 0.51923900 -1.10764400

C 0.09228700 1.73343400 -0.46370800

C 1.58920600 -0.42999300 -0.30797100

C 1.48942900 -0.52848600 1.08080700

C 2.46352700 -1.28843000 -0.97813000

C 2.24853200 -1.45112000 1.78910300

H 0.82199300 0.13383900 1.62018300

C 3.23092600 -2.21272000 -0.28101800

H 2.53506600 -1.22920300 -2.05905400

C 3.12370200 -2.29514600 1.10809700

H 2.18049500 -1.53124900 2.86811100

H 3.91114500 -2.87405400 -0.81282600

H 2.93147300 1.77202000 -1.54995900

O 3.84892800 -3.17780100 1.84658600

H 4.40771100 -3.69869700 1.25960500

C -1.08271300 1.49718200 0.43516800

H -0.83322800 1.52749300 1.50701500

H 3.50458600 3.21688500 -1.47672000

O 1.48817000 0.94293600 -2.22541200

H 2.02864300 2.49213600 -0.05497600

O 3.31018800 2.44201800 -0.93957700

H 0.93036300 1.64821200 -2.59394800

O 1.16143000 2.34925600 0.44980200

O -2.01716400 2.62583500 0.30337600

H -1.98371200 2.79367400 -0.65170400

H -1.04948800 3.82556300 0.90492900

O -0.21570500 4.32994700 1.13690500

H -0.20624000 5.10633800 0.56789300

H 0.74876100 3.24430700 0.74350400

**TS6**

C -1.79633700 0.16631600 0.14353900

C -1.39250400 -0.61952800 -0.93533400

C -2.91369400 -0.25241500 0.87970700

C -2.08512400 -1.77431300 -1.30139500

C -3.20183300 -2.14700800 -0.56315300

C -3.62239300 -1.39722400 0.53695300

H -1.72541700 -2.36079300 -2.13990800

H -4.48844100 -1.72492500 1.10218700

O -0.31714700 -0.28948100 -1.68577100

O -3.26242500 0.51877200 1.94070700

H -4.03303000 0.13366500 2.37214400

O -3.92634300 -3.25789000 -0.85757300

H -3.53537400 -3.69489400 -1.62210400

C 0.71823900 0.51119100 -1.07257300

C 0.08955300 1.69133900 -0.39234200

C 1.63492800 -0.41756600 -0.28832800

C 1.63501300 -0.44230600 1.10638800

C 2.44121700 -1.32669400 -0.97608200

C 2.42497800 -1.34745900 1.80425800

H 1.03063500 0.27232700 1.65342300

C 3.23637100 -2.23549800 -0.29012500

H 2.43618600 -1.31812700 -2.06119600

C 3.22828900 -2.24654800 1.10580900

H 2.43739600 -1.37084900 2.88813400

H 3.86195600 -2.93932300 -0.83447500

H 2.94517700 1.93445500 -1.59334500

O 3.98579300 -3.11174200 1.83391500

H 4.49525100 -3.66752400 1.23425800

C -1.06412700 1.41809600 0.47358900

H -0.89020800 1.50810500 1.55599300

H 3.33269200 3.41466300 -1.77785200

O 1.47287000 0.95731100 -2.19411800

H 1.87031800 2.65490900 -0.10767700

O 3.27003700 2.72071400 -1.11447600

H 0.88607600 1.62854200 -2.57831400

O 1.08844000 2.40863500 0.43965500

O -2.05795300 2.61129500 0.29720100

H -1.97015700 2.73350100 -0.66634000

H -1.33967800 3.50061900 0.77220600

O -0.42456500 4.11888200 1.18896700

H -0.34168300 4.97473500 0.75531600

H 0.40319800 3.42663300 0.87401900

**CP6**

C 2.07100800 -0.16200700 -0.39861000

C 1.44441800 -1.33675900 0.03271000

C 3.48088100 -0.14772600 -0.46015000

C 2.15272500 -2.48750500 0.35076400

C 3.54262500 -2.45461700 0.23518900

C 4.20827000 -1.29292600 -0.15269800

H 1.61694800 -3.37268700 0.67611400

H 5.28925800 -1.27622800 -0.20746200

O 0.07805700 -1.39592500 0.10202600

O 4.16008700 0.94869700 -0.84502600

H 3.66813700 1.77038000 -0.62854100

O 4.31069200 -3.53654900 0.52714900

H 3.74044500 -4.27571200 0.76499900

C -0.61592200 -0.18438200 0.34393600

C -0.07962700 0.91133100 -0.57201600

C -2.08174500 -0.47903900 0.12252100

C -2.51284000 -0.79358800 -1.16986500

C -3.00128200 -0.47176100 1.16444800

C -3.84048100 -1.09443300 -1.41927800

H -1.79363500 -0.79976700 -1.98382900

C -4.33963800 -0.77069600 0.92381100

H -2.67435300 -0.24188300 2.17203400

C -4.76065300 -1.08199300 -0.36680300

H -4.19096600 -1.33901600 -2.41541900

H -5.05497700 -0.76199400 1.74208200

H -1.51771600 1.88118800 1.79795100

O -6.05088000 -1.38010400 -0.66647800

H -6.58514500 -1.33431300 0.13414400

C 1.22632800 0.92957000 -0.86117400

H 1.64698300 1.71989500 -1.47570100

H -1.20231000 3.30365600 1.30803000

O -0.43449800 0.20403700 1.69651300

H -1.62999500 2.01846300 -0.34543300

O -1.90686900 2.63813500 1.33246800

H 0.50807200 0.39505700 1.80727300

O -0.94659500 1.85931700 -1.03966600

O 3.05938400 3.26687500 0.02599200

H 3.48253700 3.47120200 0.86478200

H 2.11196200 3.47439500 0.13206200

O 0.35566200 3.97607300 0.09789400

H 0.27833400 4.85807400 -0.28186500

H -0.05831400 3.36401800 -0.54490100

**PD-I**

C 2.11768600 0.69655100 -0.24122100

C 1.61510800 -0.58359800 0.01859700

C 3.51380800 0.83459600 -0.33301900

C 2.43518100 -1.69553100 0.15340700

C 3.81271300 -1.52125700 0.02538600

C 4.35841600 -0.25977200 -0.20653600

H 1.98931800 -2.66365900 0.35475800

H 5.43061300 -0.13156100 -0.28122900

O 0.26426600 -0.78133200 0.09646900

O 4.09385300 2.04223000 -0.56142400

H 3.43936900 2.74040400 -0.45123700

O 4.68351800 -2.55696700 0.14436500

H 4.19089700 -3.37041400 0.29839200

C -0.53126300 0.29732400 0.56362900

C -0.12182100 1.58743300 -0.14237100

C -1.97028200 -0.07068200 0.26644900

C -2.29823800 -0.67772400 -0.95059800

C -2.98614300 0.25340300 1.16295800

C -3.61597100 -0.97028500 -1.25999900

H -1.50495900 -0.93050600 -1.64611300

C -4.31429900 -0.03479900 0.85734200

H -2.72995500 0.71428100 2.11036800

C -4.63054000 -0.64905200 -0.35287500

H -3.88693300 -1.44813400 -2.19457900

H -5.10185000 0.21165400 1.56478300

O -5.90204700 -0.95841800 -0.70991500

H -6.50768400 -0.70307700 -0.00511400

C 1.16342100 1.77578900 -0.46928700

H 1.45278200 2.70600200 -0.94980800

O -0.39874100 0.45708800 1.94687100

H -1.93559800 2.15941200 -0.15699500

H 0.44826800 0.89145600 2.11384700

O -1.06221400 2.53861900 -0.32989700

**CP4-3**

C 2.05102700 0.13085000 -0.15718400

C 1.39658600 -1.06381800 0.14362200

C 3.45503000 0.09829100 -0.31148600

C 2.07854100 -2.26326600 0.31637900

C 3.46326300 -2.25619100 0.17129200

C 4.15863300 -1.07740200 -0.14586200

H 1.52407900 -3.16626700 0.54423700

H 5.23698500 -1.12105000 -0.25173000

O 0.04172900 -1.11493500 0.26001800

O 4.02814600 1.28106900 -0.61607000

H 4.97854600 1.17336500 -0.74549900

O 4.21455300 -3.35879200 0.31686300

H 3.66782100 -4.12474900 0.52963400

C -0.71345700 -0.01571900 -0.01224200

C -0.14832500 1.16846600 -0.33894000

C -2.15564700 -0.29783400 -0.00365500

C -2.62138200 -1.54217100 -0.45931900

C -3.08326800 0.63834800 0.47023600

C -3.97174100 -1.83157500 -0.46328600

H -1.90981600 -2.28050600 -0.81245500

C -4.44245300 0.35388800 0.47078700

H -2.74215100 1.58498700 0.87933700

C -4.89276200 -0.88192400 -0.00108500

H -4.34631400 -2.78488100 -0.81776600

H -5.15155700 1.08235300 0.85296600

O -6.19167900 -1.22807600 -0.03396400

H -6.74679500 -0.52022000 0.31418000

C 1.30328200 1.38038300 -0.31280800

H 1.66946800 2.03417400 -1.10692900

O 1.65944300 2.32090000 0.91710500

H 1.21737200 3.27496400 0.74629600

O -0.87846400 2.27545300 -0.72155800

H -1.73126100 1.98953600 -1.08332700

H 1.35232700 1.90669500 1.74042900

O 0.56673300 4.37404400 0.20516100

H 0.44535800 5.23735000 0.61488700

H -0.25172600 4.07499400 -0.22339300

**TS6**

C 2.04151300 0.11404700 -0.18019000

C 1.38323300 -1.08339600 0.11775100

C 3.45215100 0.08273100 -0.31457400

C 2.05858200 -2.28436400 0.29608000

C 3.44533200 -2.27439300 0.16683100

C 4.14757800 -1.09454000 -0.14133900

H 1.50258700 -3.18789700 0.51732800

H 5.22708000 -1.14280000 -0.23207100

O 0.02977200 -1.12109800 0.21740000

O 4.02731100 1.26294100 -0.60835000

H 4.98224400 1.16224700 -0.70748200

O 4.19519500 -3.37448600 0.31874600

H 3.64945200 -4.14258600 0.52727100

C -0.72121600 -0.02138000 -0.03952800

C -0.14073300 1.16546900 -0.36385300

C -2.16192500 -0.29289700 -0.02059100

C -2.63864700 -1.54484700 -0.44532600

C -3.08071900 0.66094500 0.43850600

C -3.99026500 -1.82530500 -0.43412200

H -1.93492600 -2.29525500 -0.78846000

C -4.44037600 0.38398200 0.45631100

H -2.73174000 1.61442000 0.82382800

C -4.90183200 -0.85955600 0.01444600

H -4.37362000 -2.78317100 -0.76606900

H -5.14226200 1.12495600 0.82743000

O -6.20186500 -1.19768700 -0.00175200

H -6.75127800 -0.47949100 0.33445200

C 1.29358200 1.33600000 -0.33343300

H 1.70442900 2.08456100 -1.00836000

O 1.65757900 2.31578700 1.08211900

H 1.29107000 3.24690600 0.86531000

O -0.85946800 2.28161600 -0.72470600

H -1.70625900 2.01069600 -1.11025800

H 1.19887700 1.94693800 1.85238300

O 0.66899400 4.40709200 0.14788700

H 0.61684300 5.33328100 0.40393800

H -0.15019000 4.13890400 -0.29179100

**PD-II**

C 2.18729800 0.45474000 -0.45596700

C 1.63978200 -0.78779200 -0.07611200

C 3.60999200 0.58883400 -0.42374000

C 2.40877500 -1.87486700 0.31217700

C 3.78856700 -1.69658700 0.33473900

C 4.39082100 -0.46970400 -0.03338900

H 1.93539900 -2.80715100 0.59573000

H 5.47250300 -0.40139500 0.00581400

O 0.30180700 -0.94894000 -0.11517400

O 4.07923400 1.78816900 -0.79733900

H 5.04411800 1.80950600 -0.76499300

O 4.63853800 -2.65844900 0.70237600

H 4.17347900 -3.47034600 0.94100400

C -0.54237500 0.03213500 -0.40261500

C -0.04475400 1.26462000 -0.84842000

C -1.94473100 -0.32433100 -0.29790100

C -2.36572100 -1.62852100 -0.62089500

C -2.88284600 0.60772000 0.18003400

C -3.69357300 -1.98068200 -0.51245300

H -1.64076800 -2.35398400 -0.97450000

C -4.21498300 0.24920700 0.30414100

H -2.55637300 1.58497900 0.52864700

C -4.62908300 -1.04020600 -0.05236600

H -4.04292100 -2.97258300 -0.77404800

H -4.93467600 0.96143500 0.69670200

H -0.15592100 1.75458100 1.98433900

O -5.90207400 -1.44814100 0.03020500

H -6.47447400 -0.74455900 0.36035000

C 1.32219000 1.46716000 -0.87798700

H 1.71868900 2.41214900 -1.23663200

H -1.26975500 4.04886300 1.85713400

O 0.29837400 0.89682000 2.02398200

H -0.99911100 3.45386500 0.45153800

O -1.17459300 3.22227000 1.37281600

H 0.23507100 0.60806900 2.93902000

O -0.86080100 2.27581700 -1.25082200

H -1.72529000 1.92584700 -1.51393400

**TS7**

C 2.15774000 0.42863000 -0.48607400

C 1.71606900 -0.65772900 0.27079800

C 3.53132000 0.50045300 -0.79148500

C 2.56294400 -1.65532500 0.72279600

C 3.91943900 -1.55455100 0.39792300

C 4.40775400 -0.47753500 -0.35048700

H 2.16555900 -2.48760800 1.29271200

H 5.46801700 -0.43761100 -0.57488400

O 0.37446900 -0.81179100 0.55975200

O 3.91660200 1.56635500 -1.52337900

H 4.85841400 1.51402500 -1.72648700

O 4.82467900 -2.46773000 0.78806900

H 4.40270600 -3.18708800 1.27267900

C -0.50208500 0.26473000 0.47940500

C -0.06768500 1.29897200 -0.53010700

C -1.89740200 -0.24159600 0.19169500

C -2.12332100 -0.83172400 -1.06093900

C -2.95146700 -0.12562400 1.09361700

C -3.37949000 -1.29869300 -1.40351900

H -1.29871100 -0.94188200 -1.76142700

C -4.21698200 -0.59542100 0.75710900

H -2.79049000 0.32039100 2.06813000

C -4.43627100 -1.18354100 -0.49005400

H -3.57015500 -1.76596800 -2.36272500

H -5.03375000 -0.50755700 1.46763600

H -0.83761000 2.25129100 1.76651000

O -5.63270600 -1.65602500 -0.88377700

H -6.28974800 -1.55755100 -0.18437400

C 1.18932000 1.38936900 -0.96457100

H 1.49032200 2.15896300 -1.66784600

H -0.71766600 3.96702300 1.63968100

O -0.50888400 0.91193300 1.81181100

H -1.26046100 3.01551500 0.37679100

O -1.23534600 3.18412800 1.40274800

H 0.18346900 0.51464200 2.35980500

O -1.03926700 2.24695600 -0.87507500

H -1.82557400 1.79032200 -1.22402500

**CP7**

C 2.19628400 0.48887600 -0.43574100

C 1.73959000 -0.67787600 0.18340300

C 3.58028800 0.60653100 -0.67593800

C 2.58315200 -1.70851800 0.56220100

C 3.94933800 -1.55973600 0.30485500

C 4.45174200 -0.40379100 -0.30482700

H 2.17510300 -2.59782100 1.02893700

H 5.51951100 -0.32987400 -0.47956500

O 0.39139700 -0.86962900 0.39886200

O 3.97821700 1.74722600 -1.27605000

H 4.92756100 1.72569000 -1.44742600

O 4.85218600 -2.49837600 0.63390100

H 4.42284400 -3.26908900 1.02404400

C -0.47174000 0.21800000 0.43853300

C -0.03899500 1.33250400 -0.48547400

C -1.87949800 -0.24690000 0.15428800

C -2.18289000 -0.61254600 -1.16563000

C -2.86587000 -0.33542800 1.13221500

C -3.45263100 -1.04452900 -1.50458300

H -1.40463500 -0.58282800 -1.92613300

C -4.14435800 -0.77163500 0.79976600

H -2.64241600 -0.08033200 2.16165800

C -4.44411800 -1.12516000 -0.51716300

H -3.70217800 -1.33875300 -2.51747500

H -4.90851300 -0.84253600 1.56833900

H -1.06339200 1.97106400 1.84816400

O -5.65835600 -1.54950800 -0.91040300

H -6.26613300 -1.59793300 -0.16281400

C 1.23188600 1.47703100 -0.86492200

H 1.54678400 2.30875100 -1.48665400

H -1.30238500 3.68855000 1.94502700

O -0.45402300 0.73918500 1.82943700

H -1.46701800 2.84181700 0.50711200

O -1.59817500 2.86183400 1.53797200

H 0.46114300 0.70147900 2.14967400

O -1.01911800 2.28536200 -0.79634600

H -1.74637000 1.85213200 -1.27776700


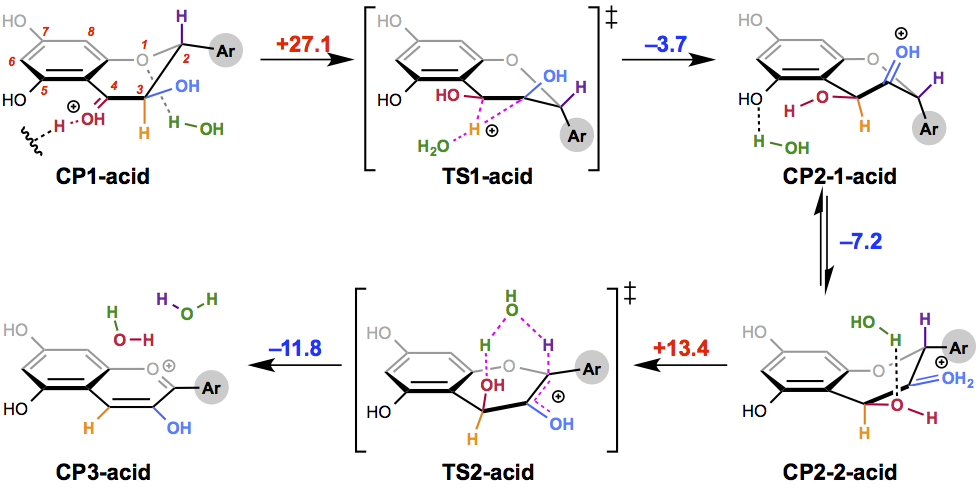
**3.2 Hypothetical Acid-Catalyzed Enzymatic Reaction**

**Figure S1. Calculation results for the conversion of dihydroflavonol (CP1-acid) to 2-flaven-3,4-diol (CP3-acid) *via* route B in an acidic aqueous media.**

**CP1-acid**

C -0.76085300 1.61623300 0.36622400

C -1.49962600 0.44492100 0.21324500

C -1.37022300 -0.28212300 -1.01708900

C -2.47511800 -0.00739300 1.15784600

C -2.19339900 -1.35856000 -1.29138200

C -3.13485400 -1.75509400 -0.34014500

C -3.27588500 -1.08870500 0.90092800

H -2.10173100 -1.89662000 -2.22595600

H -4.01138800 -1.42369500 1.62578600

O -0.48031900 0.05086700 -1.94745500

O -0.64867300 2.91566400 -1.58104400

H -0.75504100 3.78023300 -1.16490000

C 0.61518800 0.90487600 -1.59930300

C 0.13092400 2.08351800 -0.75897400

C 1.72795100 0.12056500 -0.93228800

C 1.58820900 -1.22155500 -0.57584600

C 2.93404900 0.76548500 -0.65291300

C 2.61070100 -1.89161300 0.08722800

H 0.68044900 -1.76454900 -0.81538700

C 3.96616900 0.10643100 0.00198200

H 3.08394300 1.80003400 -0.95258500

C 3.80636200 -1.22983200 0.38190100

H 2.51460300 -2.93549200 0.36368300

H 4.89943000 0.62350800 0.20643300

H 0.94722300 1.31713000 -2.55615300

O 4.75808700 -1.93473600 1.02717400

H 5.56617400 -1.41598700 1.11735200

O -0.88164600 2.47874300 1.32292700

H -1.48188300 2.15047100 2.02165200

H 1.58878200 1.01936100 2.59539900

H 1.48264900 -0.21230100 1.69289500

H 0.99310700 2.59565400 -0.31761800

O 0.98168500 0.54922800 2.01404800

O -3.88628800 -2.80282000 -0.65445700

H -4.51201000 -3.02984900 0.04565400

O -2.55335700 0.71458300 2.29571900

H -3.22778200 0.37695200 2.89997800

**TS1-acid**

C -0.73450300 1.76053700 0.15541200

C -1.57559100 0.54047800 0.06422500

C -1.46843300 -0.28971100 -1.05898400

C -2.50797300 0.19272000 1.04834400

C -2.28602700 -1.38973000 -1.24287600

C -3.23265200 -1.68866000 -0.25970400

C -3.34128600 -0.90615700 0.89575600

H -2.19511900 -2.00901200 -2.12604900

H -4.06694800 -1.15369000 1.66516300

O -0.55431500 -0.02318900 -2.04384600

O 0.77901600 3.08999100 -1.02531800

H 1.49631400 3.06594900 -1.67561600

C 0.59517100 0.71661900 -1.69912600

C 0.20699700 1.92547600 -0.86245300

C 1.66318000 -0.09039700 -0.97722300

C 1.39449800 -1.35060500 -0.43815700

C 2.91843100 0.48356400 -0.76762000

C 2.35564100 -2.00959600 0.31947600

H 0.43690400 -1.82936600 -0.60890600

C 3.88942500 -0.16768200 -0.01776800

H 3.16498500 1.45333500 -1.19509100

C 3.61011000 -1.42435900 0.53286800

H 2.16704700 -2.99328200 0.73446900

H 4.86579300 0.28652600 0.12374600

H 0.98854800 1.09198400 -2.65231300

O 4.49343400 -2.12167800 1.26956200

H 5.35074600 -1.67990900 1.29805400

O -1.15645800 2.88225100 0.79475600

H -1.74080400 2.61452100 1.52151200

H 1.65174900 1.39803400 2.45046900

H 1.65869500 0.01288200 1.73304100

H 0.32810600 1.35516000 0.72536400

O 1.10616000 0.78056300 1.94810700

O -4.00287300 -2.76548700 -0.47819700

H -4.63066100 -2.90070100 0.24157000

O -2.55587900 0.99918300 2.14401800

H -3.26995700 0.73952000 2.73891400

**CP2-1-acid**

C -0.63208800 1.72194500 0.46110500

C -1.46783000 0.49598300 0.15588600

C -1.43249500 -0.14580300 -1.07947400

C -2.31805900 -0.01917300 1.13632600

C -2.24255300 -1.22819900 -1.37820800

C -3.11479500 -1.69346700 -0.39226600

C -3.14870800 -1.10057700 0.87600500

H -2.20575300 -1.70409400 -2.34988900

H -3.81415200 -1.48119300 1.64582800

O -0.58489200 0.29306100 -2.07594100

O 0.43285000 3.30923700 -0.90006100

H 0.95853100 3.47400800 -1.70474300

C 0.56784000 0.99453500 -1.67065000

C 0.13918200 2.08434500 -0.72244400

C 1.62141200 0.14733500 -0.97193600

C 1.46305200 -1.23370500 -0.84315600

C 2.76522400 0.76062400 -0.45801900

C 2.43484700 -1.98756900 -0.19894600

H 0.58750300 -1.72511100 -1.25282100

C 3.74193100 0.01586300 0.18675100

H 2.91267000 1.83524700 -0.55063300

C 3.58006800 -1.36727100 0.31924100

H 2.34083800 -3.06345000 -0.10309500

H 4.62518500 0.50436500 0.58635600

H 0.97158700 1.44285600 -2.58811400

O 4.47671700 -2.15958000 0.93100900

H 5.23901800 -1.65245300 1.23564600

O -1.29705800 2.83279400 0.96066900

H -1.70467800 2.56596700 1.79627700

H 0.61787600 -0.27628500 2.98472700

H 1.39367600 -0.74058300 1.73635900

H 0.18353600 1.35435400 1.14873800

O 1.08472700 0.04488900 2.20602500

O -3.88822300 -2.74184600 -0.72174500

H -4.46865900 -2.99163200 0.00680800

O -2.27544400 0.61026500 2.34486900

H -2.96875700 0.27826200 2.92864800

**CP2-2-acid**

C 1.65753100 0.26924500 -0.51828100

C 1.39636100 -0.88751700 0.21736900

C 2.96387300 0.48426200 -0.96940800

C 2.38532100 -1.82002000 0.50034900

C 3.67700200 -1.58493200 0.02374000

C 3.97419300 -0.42468800 -0.70059100

H 2.13205800 -2.70560500 1.07232400

H 4.98860700 -0.27029400 -1.05209200

O 0.12523500 -1.17380000 0.64584100

O 3.17900900 1.62626300 -1.68350100

H 4.09337300 1.68330800 -1.98719600

O 4.69946800 -2.42810500 0.24319600

H 4.40363900 -3.21810800 0.71147000

C -0.72931100 -0.12552500 1.05483800

C -0.32000500 1.16709400 0.46167200

C -2.13359900 -0.33974300 0.50552100

C -2.32867800 -1.05874600 -0.67838100

C -3.22481200 0.23346700 1.15940800

C -3.60403600 -1.22015100 -1.19086500

H -1.48327400 -1.52437100 -1.17515600

C -4.50490400 0.07956500 0.64851900

H -3.08077700 0.79507300 2.07850300

C -4.69908000 -0.64902200 -0.53070200

H -3.78381100 -1.78986200 -2.09509700

H -5.35438500 0.51413600 1.16697000

H -0.72728000 -0.02927200 2.14552700

O -5.90927700 -0.84237100 -1.08206300

H -6.60161700 -0.42963500 -0.55191500

C 0.49617500 1.20027100 -0.79476500

H -0.14359100 0.79006900 -1.59639700

O 0.76154000 2.55421400 -1.02600800

H 1.67023100 2.66112400 -1.35310400

O -0.69890600 2.26294300 0.95819200

H -0.30361200 2.98473200 0.39864500

O 1.60473600 1.26929900 2.18745200

H 1.64223400 1.69348100 3.05229900

H 2.41758800 0.75632400 2.10981200

**TS2-acid**

C -2.08072700 -0.31718800 -0.33633600

C -1.44460700 0.90089500 -0.12993200

C -3.48130700 -0.34929800 -0.24881300

C -2.14784600 2.06500700 0.15846100

C -3.53755400 1.99660700 0.23648300

C -4.21532500 0.78310400 0.04094500

H -1.60536700 2.99021500 0.31651900

H -5.29704900 0.76793300 0.11655400

O -0.08051100 1.02060700 -0.20594900

O -4.03877600 -1.57257100 -0.47526000

H -5.00274000 -1.51733100 -0.47596900

O -4.30577700 3.06269200 0.51114600

H -3.77150400 3.85928400 0.61559500

C 0.69214600 -0.12188000 -0.19874100

C 0.14238100 -1.32794100 -0.67514900

C 2.14668100 0.15986000 -0.12970500

C 2.63469100 1.36394400 -0.65316200

C 3.02806200 -0.74262000 0.47139900

C 3.98612500 1.64923600 -0.59815800

H 1.95013200 2.07310000 -1.10476600

C 4.38404200 -0.45846500 0.53117600

H 2.65762100 -1.66661300 0.90396400

C 4.86941600 0.73694900 -0.00796000

H 4.38708700 2.57036100 -1.00451700

H 5.06612200 -1.16063700 1.00126900

H 0.38000000 -0.80756200 0.84990100

O 6.17062100 1.07595400 0.01229900

H 6.69920000 0.38793300 0.43386000

C -1.34187600 -1.54916500 -0.74745800

H -1.57940700 -1.81976700 -1.78838300

O -1.56680400 -2.68880200 0.08633600

H -2.50328600 -2.92550700 -0.00959200

O 0.90324100 -2.38142900 -0.80509400

H 0.35651300 -3.18624800 -0.73295400

O 0.07180500 -1.63637800 2.09595900

H -0.03504700 -1.20550100 2.95314800

H -0.73849300 -2.13452200 1.89158000

**CP3-acid**

C 2.10424100 0.07207400 -0.19333200

C 1.44171400 -1.15693800 -0.03722600

C 3.53270800 0.05371000 -0.20601200

C 2.09757600 -2.37360700 0.10238900

C 3.48784300 -2.34473500 0.08228300

C 4.20589600 -1.13526800 -0.07008600

H 1.53394500 -3.29207400 0.21444700

H 5.28939900 -1.18393500 -0.07708600

O 0.09319000 -1.16250000 -0.02617900

O 4.12068800 1.25027700 -0.35532900

H 5.08231400 1.16403600 -0.36353800

O 4.24066900 -3.44288100 0.20335700

H 3.69725000 -4.23501300 0.29951700

C -0.67859000 -0.07866300 -0.15707900

C -0.05171700 1.18127800 -0.30318000

C -2.09264800 -0.36988500 -0.10069900

C -2.51448600 -1.64602200 0.34251800

C -3.07471600 0.55999600 -0.50126800

C -3.84856500 -1.97514900 0.39603400

H -1.77792700 -2.37571400 0.65619200

C -4.41527100 0.22630700 -0.45972300

H -2.78321400 1.53584100 -0.86054600

C -4.81342900 -1.03816300 -0.00855000

H -4.18171700 -2.94563800 0.74432700

H -5.16166000 0.94616300 -0.78227000

H -0.51064200 3.16159500 1.78381300

O -6.09352700 -1.41921400 0.06341500

H -6.68379100 -0.71777100 -0.23936900

C 1.33451300 1.23716900 -0.31669900

H 1.82693000 2.20047800 -0.41853900

O 0.78672200 4.27192600 -0.61659800

H 0.63925000 5.12285500 -1.04192500

O -0.82536100 2.27240200 -0.39115600

H -0.26490000 3.07685700 -0.60611900

O 0.38810700 3.49700500 1.88635700

H 0.49346700 3.73011100 2.81434300

H 0.82698000 4.40346100 0.34961100

**3.3 Hypothetical Base-Catalyzed Enzymatic Reaction**


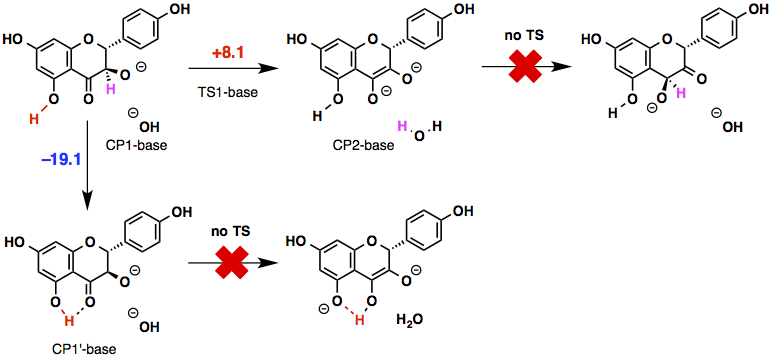


**Figure S2. Calculation results for the conversion of dihydroflavonol (CP1-base) to 2-flaven-3,4-diol (CP2-base) *via* route B in a basic aqueous media.**

**CP1-base**

C 1.42732600 -2.01502200 0.07782300

C 2.17476100 -0.70364300 0.04562600

C 1.43754000 0.49995400 0.10060000

C 3.57249200 -0.58465800 -0.01232000

C 2.04740100 1.76810700 0.07708800

C 3.42406900 1.82727300 0.03129100

C 4.19982700 0.66324900 -0.00863500

H 1.40265500 2.64195100 0.03414600

H 5.28914800 0.72721000 -0.03641200

O 0.11654100 0.50795300 0.19132700

O -0.73290700 -3.04810900 -0.15066300

C -0.62214500 -0.62263600 -0.36158700

C -0.12545900 -1.96303100 0.27500300

C -2.09645500 -0.25123000 -0.20462000

C -3.01906800 -1.27792600 0.02570000

C -2.47210200 1.11602300 -0.33841900

C -4.38242700 -0.99093400 0.15186000

H -2.63351600 -2.29500800 0.09343400

C -3.85077700 1.33507000 -0.19648600

H -1.44826700 2.47128500 -0.28604100

C -4.79124600 0.32976900 0.03811700

H -5.11848700 -1.77112600 0.33401400

H -4.24872200 2.36249000 -0.26724600

H -0.36587100 -0.65418000 -1.43559500

O 2.03156000 -3.06620700 -0.02447000

H 5.26503200 -1.41108800 -0.06998300

H -0.57792400 2.94377300 0.86420600

H -0.17569400 -1.72786500 1.39932500

O -0.86098500 3.30621400 0.01640900

O 4.03243300 3.06247300 0.00922200

H 4.97640500 2.91367500 -0.10535900

O 4.34923100 -1.70930300 -0.05231500

O -6.14935100 0.62612300 0.16188800

H -6.19937200 1.58356000 0.05806600

**TS1-base**

C -1.28266700 1.81435100 -0.30213900

C -2.06438800 0.51187200 -0.16249400

C -1.41489400 -0.73937100 -0.28471900

C -3.45497100 0.45402900 0.02879900

C -2.09929200 -1.96323800 -0.21804200

C -3.46880000 -1.96190300 -0.02834500

C -4.15693700 -0.76025500 0.08743400

H -1.54596200 -2.88935100 -0.31537100

H -5.23952900 -0.75667500 0.23290300

O -0.09371600 -0.85595600 -0.47258800

O 1.02494400 2.62310200 -0.39324100

C 0.65447800 0.35404500 -0.80307200

C 0.17397900 1.65425300 -0.11775400

C 2.09849500 0.02924600 -0.47828200

C 3.00113700 -0.29221700 -1.48669300

C 2.52792400 -0.01484700 0.85760700

C 4.32563300 -0.64558900 -1.20899200

H 2.66578900 -0.25580100 -2.52170700

C 3.84280400 -0.36891800 1.13653200

H 1.78605700 0.23424100 1.65482200

C 4.74030400 -0.68041300 0.11329100

H 5.03365000 -0.88930800 -1.99641400

H 4.18979000 -0.40131600 2.17206200

H 0.58180300 0.48123400 -1.89967000

O -1.90031500 2.88116100 -0.40274100

H -5.06783200 1.34145400 0.42467700

H -0.20561800 -0.17453900 2.23681100

H 0.08086900 1.28308500 1.12814400

O 0.23264100 0.65241000 2.48797900

O -4.13233900 -3.17628500 0.02561000

H -5.05394400 -2.97688400 0.21863700

O -4.17492200 1.61046800 0.18394800

O 6.05374600 -1.03403700 0.38676000

H 6.15778200 -0.94930700 1.34050700

**CP2-base**

C 0.76989700 1.87474700 0.39983600

C 1.47656100 0.62338800 0.13938700

C 1.38269800 -0.50557400 1.00759000

C 2.36704200 0.40023000 -0.95801800

C 2.09664400 -1.68838900 0.85182900

C 2.97658500 -1.82872600 -0.21538800

C 3.09562900 -0.77950600 -1.12075200

H 1.94542400 -2.48126500 1.58319300

H 3.75991000 -0.89447600 -1.97751100

O 0.57036300 -0.44543100 2.11185000

O -1.02550000 2.70701600 1.78973000

C -0.55882400 0.41146400 1.92801100

C -0.21740100 1.80640300 1.40894000

C -1.57738100 -0.24872000 0.99902500

C -1.76847600 -1.62950500 1.00418500

C -2.26424100 0.52777000 0.05732300

C -2.64547300 -2.24775900 0.10287100

H -1.19529400 -2.23043800 1.70573600

C -3.14688700 -0.07899300 -0.83366900

H -2.07976400 1.59858200 0.00486900

C -3.33467600 -1.46269900 -0.80928700

H -2.77942200 -3.32574800 0.07996900

H -3.66798100 0.52930100 -1.57315300

H -0.99126600 0.52497900 2.93003300

O 0.97688100 2.96057800 -0.31598400

H 3.07408600 0.99945600 -2.59750200

H -0.83179000 1.84431300 -2.00010000

H -0.24242300 3.00034300 -1.22098900

O -1.02485600 2.78076900 -1.87446000

O 3.72484600 -2.99389800 -0.41852000

H 3.33425100 -3.65286100 0.16396500

O 2.50935200 1.38179700 -1.91680900

O -4.19906900 -2.08527600 -1.69606700

H -4.49205900 -1.40141600 -2.30773100

**CP1'-base**

C -1.43227000 -1.93226000 -0.09854100

C -2.15147800 -0.65261400 -0.05463600

C -1.43289600 0.55610500 -0.10428100

C -3.56254800 -0.61305000 -0.00141200

C -2.09050000 1.79444800 -0.08062900

C -3.47668300 1.79120500 -0.03500100

C -4.23029000 0.61170400 0.00048100

H -1.49287800 2.69911000 -0.03369500

H -5.31704500 0.62827300 0.03002600

O -0.11050300 0.56207700 -0.19415100

O 0.67552900 -3.00802700 0.29448900

C 0.61354100 -0.57249400 0.38499400

C 0.11320400 -1.93339200 -0.21179200

C 2.09280300 -0.23257100 0.21598100

C 2.99039900 -1.27417600 -0.04322800

C 2.49407700 1.12588200 0.36304900

C 4.35611400 -1.01144500 -0.19158800

H 2.59356500 -2.28693000 -0.10266500

C 3.87410500 1.31974900 0.19773400

H 1.45843500 2.51432400 0.32533800

C 4.79069400 0.30039900 -0.06955900

H 5.07409300 -1.80295900 -0.39462000

H 4.29473400 2.33743000 0.27627700

H 0.35439400 -0.57661000 1.45778300

O -2.08978800 -2.98852300 -0.05928800

H -3.52528300 -2.47259500 0.00691100

H 0.54531500 2.94520000 -0.79763400

H 0.21656800 -1.75589100 -1.34367900

O 0.86755500 3.33270000 0.02436400

O -4.11811600 3.00771700 -0.00951600

H -5.05963800 2.82702200 0.07887900

O -4.25714900 -1.75933800 0.03128700

O 6.15067000 0.57237400 -0.21636600

H 6.22252100 1.52684600 -0.09889600

**REFERENCES**

1. M. J. Frisch, G. W. Trucks, H. B. Schlegel, G. E. Scuseria, M. A. Robb, J. R. Cheeseman, G. Scalmani, V. Barone, B. Mennucci, G. A. Petersson, H. Nakatsuji, M. Caricato, X. Li, H. P. Hratchian, A. F. Izmaylov, J. Bloino, G. Zheng, J. L. Sonnenberg, M. Hada, M. Ehara, K. Toyota, R. Fukuda, J. Hasegawa, M. Ishida, T. Nakajima, Y. Honda, O. Kitao, H. Nakai, T. Vreven, J. A. Montgomery, Jr., J. E. Peralta, F. Ogliaro, M. Bearpark, J. J. Heyd, E. Brothers, K. N. Kudin, V. N. Staroverov, R. Kobayashi, J. Normand, K. Raghavachari, A. Rendell, J. C. Burant, S. S. Iyengar, J. Tomasi, M. Cossi, N. Rega, J. M. Millam, M. Klene, J. E. Knox, J. B. Cross, V. Bakken, C. Adamo, J. Jaramillo, R. Gomperts, R. E. Stratmann, O. Yazyev, A. J. Austin, R. Cammi, C. Pomelli, J. W. Ochterski, R. L. Martin, K. Morokuma, V. G. Zakrzewski, G. A. Voth, P. Salvador, J. J. Dannenberg, S. Dapprich, A. D. Daniels, Ö. Farkas, J. B. Foresman, J. V. Ortiz, J. Cioslowski, and D. J. Fox, Gaussian, Inc., Wallingford CT, 2009.

2. S. Maeda, Y. Osada, K. Morokuma, K. Ohno, **2012**, GRRM 11 Version 11.03.

3. S. Maeda, K. Ohno, K. Morokuma, *Phys. Chem. Chem. Phys.* **2013**, *15*, 3683–3701.

4. K. Ohno, S. Maeda, *Chem. Phys. Lett.* **2004**, *384*, 277–282.

5. S. Maeda, K. Ohno, *J. Phys. Chem* *A*. **2005**, *109*, 5742–5753.

6. K. Ohno, S. Maeda, *J. Phys. Chem A.* **2006**, *110*, 8933–8941.

7. Y. Zhao, D. G. Truhlar, *Theor. Chem. Acc.* **2008***,* *120*, 215–241.

8. W. J. Hehre, R. Ditchfield, J. A. Pople, *J. Chem. Phys.* **1972**, *56*, 2257–2261.

9. M. M. Francl, *et al.* *J. Chem. Phys.* **1982**, *77*, 3654–3665.

10. R. Krishnan, J. S. Binkley, R. Seeger, J. A. Pople, *J. Chem. Phys.* **1980**, *72*, 650–654.

11. A. D. McLean, G. S. Chandler, *J. Chem. Phys.* **1980**, *72*, 5639–5648.

12. S. Miertuš, E. Scrocco, J. Tomasi, *Chem. Phys.* **1981**, *55*, 117–129.

13. J. L. Pascual-Ahuir, E. Silla, I. Tuñón, *J. Comp. Chem.* **1994**, *15*, 1127–1138.

14. E. Cancès, B. Mennucci, J. Tomasi, *J. Chem. Phys.* **1997**, *107*, 3032–3041.

15. M. Cossi, V. Barone, B. Mennuci, J. Tomasi, *Chem. Phys. Lett.* **1998**, *286*, 253–260.

16. J. Tomasi, B. Mennucci, E. Cancès, *J. Mol. Struct.* (*THEOCHEM*) **1999**, *464*, 211-226.

17. J. Tomasi, B. Menucci, R. Cammi, *Chem. Rev.* ***2005****,* *105*, 2999–3093.
